# Mapping the ‘early salinity response’ triggered proteome adaptation in contrasting rice genotypes using iTRAQ approach

**DOI:** 10.1186/s12284-018-0259-5

**Published:** 2019-01-30

**Authors:** Nita Lakra, Charanpreet Kaur, Sneh Lata Singla-Pareek, Ashwani Pareek

**Affiliations:** 1 Stress Physiology and Molecular Biology Laboratory, School of Life Sciences, Jawaharlal Nehru University, New Delhi, 110067 India; 2 Plant Stress Biology, International Centre for Genetic Engineering and Biotechnology, Aruna Asaf Ali Road, New Delhi, 110067 India

**Keywords:** iTRAQ, Proteomics, Pokkali, Rice, Salinity, Seedlings

## Abstract

**Background:**

To delineate the adaptive mechanisms operative under salinity stress, it is essential to study plant responses at the very early stages of stress which are very crucial for governing plant survival and adaptation. We believe that it is the initial perception and response phase which sets the foundation for stress adaptation in rice seedlings where plants can be considered to be in a state of osmotic shock and ion buildup.

**Results:**

An isobaric Tags for Relative and Absolute Quantitation (iTRAQ) approach was used to analyze the pre-existing differences as well as the very early salt shock responsive changes in the proteome of seedlings of contrasting rice genotypes, viz salt-sensitive IR64 and salt-tolerant Pokkali. In response to a quick salt shock, shoots of IR64 exhibited hyperaccumulation of Na^+^, whereas in Pokkali, these ions accumulated more in roots. Interestingly, we could find 86 proteins to be differentially expressed in shoots of Pokkali seedlings under non-stress conditions whereas under stress, 63 proteins were differentially expressed in Pokkali shoots in comparison to IR64. However, only, 40 proteins under non-stress and eight proteins under stress were differentially expressed in Pokkali roots. A higher abundance of proteins involved in photosynthesis (such as, oxygen evolving enhancer proteins OEE1 & OEE3, PsbP) and stress tolerance (such as, ascorbate peroxidase, superoxide dismutase, peptidyl-prolyl cis-trans isomerases and glyoxalase II), was observed in shoots of Pokkali in comparison to IR64. In response to salinity, selected proteins such as, ribulose bisphosphate carboxylase/oxygenase activase, remained elevated in Pokkali shoots. Glutamate dehydrogenase - an enzyme which serves as an important link between Krebs cycle and metabolism of amino acids was found to be highly induced in Pokkali in response to stress. Similarly, other enzymes such as peroxidases and triose phosphate isomerase (TPI) were also altered in roots in response to stress.

**Conclusion:**

We conclude that Pokkali rice seedlings are primed to face stress conditions where the proteins otherwise induced under stress in IR64, are naturally expressed in high abundance. Through specific alterations in its proteome, this proactive stress machinery contributes towards the observed salinity tolerance in this wild rice germplasm.

**Electronic supplementary material:**

The online version of this article (10.1186/s12284-018-0259-5) contains supplementary material, which is available to authorized users.

## Backgound

Soil salinity is one of the major hurdles faced by agricultural scientists throughout the world as it severely limits crop productivity and yield (Pareek et al. 2010; Joshi et al. 2018). Excessive accumulation of Na^+^ in the soil inhibits absorption of moisture and mineral nutrients, resulting in buildup of toxic ions and reactive oxygen species (ROS) in plants (Kim et al. 2008). These toxic byproducts can diminish enzyme activity or even degrade cellular proteins. To overcome this, plants have acquired dynamic responses at various levels to facilitate their survival under stress (Munns and Tester 2008). In plants, salinity triggers the expression of genes that function in both salt response and in increasing salt tolerance. Though all plants try to adjust to these unforeseen situations, but it is the timely and well-coordinated response acquired in selected genotypes which leads to their successful adaptation and hence, survival under stress (Lakra et al. 2018). On the other hand, susceptible species succumb to stress due to their inability to efficiently channelize resources towards stress management. An understanding of the behavior of both tolerant and susceptible species is thus, worth investigating to identify differences at molecular, physiological and biochemical levels under stress conditions which can then be employed to engineer stress tolerance in plants.

Rice (*Oryza sativa* L.) is a major cereal crop and is a key source of dietary starch for about half of the population (Fageria 2007). Its growth and productivity is significantly affected by salinity conditions and thus, rice is considered to be salt-sensitive with degree of its sensitivity varying during different growth phases (Moradi and Ismail 2007). The cultivation practice for rice known as SRI (System of Rice Intensification), involves transplantation of young seedlings (8–12 days old; with 2–3 leaf stage) to the field so as to preserve their potential for tillering and rooting ability. Thus, this seedling stage is considered to be very important for governing the fate of successful crop production.

IR64 is a semi-dwarf and high yielding, variety of *indica* rice which is moderately sensitive to salinity whereas Pokkali, another *indica* rice having a high protein content, medicinal property, high amylase content (good for the diabetic patients) and peculiar taste (Agriculture Department, Government of Kerala; http://sites.cdit.org/wto/index.php/pokkali-rice), is a wild landrace with unique saline tolerant genes (Thomson et al. 2010; Waziri et al. 2016; Nutan et al. 2017). It is cultivated in an organic way accompanied by integrated farming with prawn culture in the water logged coastal regions of Kerala in Southern India inundated with saline sea water in the field (Vijayan 2016). These two genotypes, owing to differences in their response to salt stress, have been extensively used as research material by plant scientists. Gene expression studies have revealed that salt tolerance of Pokkali may be due to constitutively high expression of several genes that contribute to salinity tolerance, such as CaMBP, glutathione transferases, late embryogenesis abundant proteins, V-ATPase, OSAP1 zinc finger protein and HBP1B transcription factor (TF) but these are stress inducible in IR64 (Kumari et al. 2009; Soda et al. 2013). Interestingly, recent RNA seq-based transcriptome studies in IR64 (salt sensitive), Pokkali (salt-tolerant) and N22 (drought-tolerant) genotype have revealed that a total of 801 transcripts in N22 and 507 in Pokkali to be exclusively differentially expressed under stress conditions (Shankar et al. 2016). Gene ontology studies further suggested an enrichment of transcripts involved in abiotic stress response and regulation of gene expression in these stress-tolerant rice cultivars. Specifically, members of bHLH and C_2_H_2_ transcription families in Pokkali exhibited differential regulation under salinity and desiccation stresses, respectively and transcripts involved in wax and terpenoid metabolism were also found to be up-regulated. However, at proteome level, not much is still known about the differential response of these genotypes. Proteome studies in other rice varieties though have been carried out. For instance, Xu et al. (2015) have studied changes in protein profiles in *Japonica* rice cultivar Zhonghua11 (ZH11) after 24 h of salinity stress and identified 56 proteins to be differentially regulated with 16 of them being enriched in antioxidant pathways, oxidative phosphorylation and photosynthesis. Further, comparative proteome analysis of two contrasting African rice genotypes has also been carried out indicating proteins involved in redox homeostasis, stress, and signaling categories to be differentially responsive in the sensitive and tolerant genotypes (Damaris et al. 2016).

**Fig. 1:**
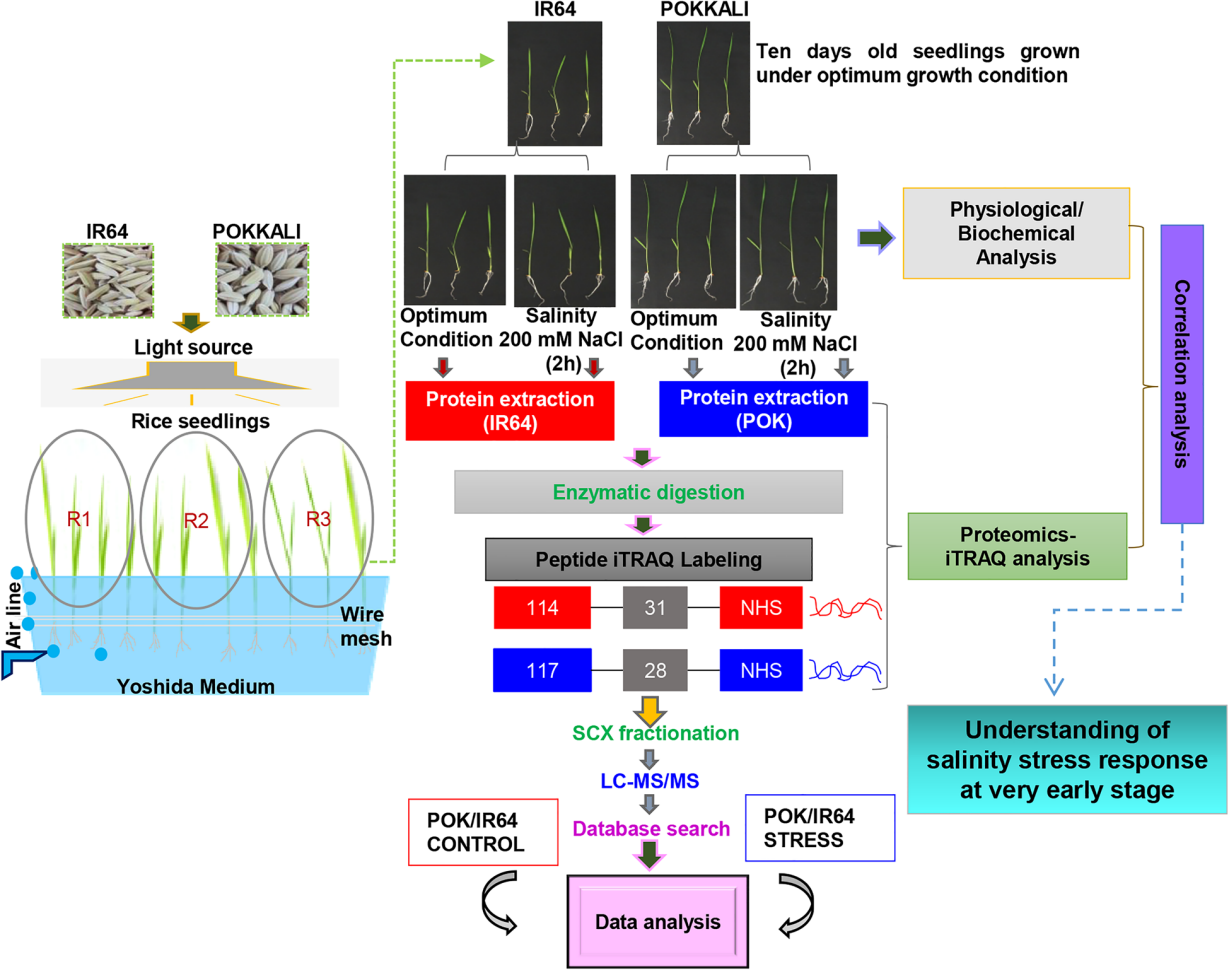
Flowchart depicting the experimental steps involved in the present study. Ten-day old rice seedlings of Pokkali and IR64 were subjected to salinity stress (200 mM NaCl) for a period of 2 h. To understand the mechanism of tolerance/sensitivity, shoot and root tissues were used for physio-chemical and proteome studies under control and salinity conditions. For the proteome studies, the protein extracts were trypsin digested and peptides were labeled with isobaric tags for iTRAQ reagents. For the peptide labeling, different iTRAQ labels (represented as 114 and 117; reporters with balance group 31 and 28 respectively) were used in this study. Labeled peptides were fractionated by strong cation exchange chromatography (SCX) and analyzed using LC-MS/MS (Liquid chromatography–mass spectrometry). POK represents Pokkali genotype and R1, R2 and R3 represent the replicates 1, 2 and 3 respectively

Recently, we have reported a comparative temporal proteome analysis of Pokkali and IR64 genotypes in response to salinity stress using 2D-DIGE (Lakra et al. 2018). Our findings suggested that Pokkali proteome exhibits increased expression of photosynthesis-related proteins after 15 min and 2 h of salinity stress in contrast to IR64 which shows greater perturbations in metabolism-related proteins during this phase. However, at later stages viz. 24–72 h, stress acclimation response is induced in Pokkali, and at this time, proteins which were found to be early induced in Pokkali can be seen to be induced in IR64, suggesting a late induction of stress response in IR64. The study thus indicated towards important differences in the cellular machinery of these genotypes and suggested that early stress response forms an important component of plant stress adaptation machinery which needs to be studied in details to decipher the basis of stress tolerance in plants. Therefore, in the present work, we have studied the response of rice seedlings to very early stress, a state where plants can be considered to be in a state of osmotic shock in response to stress. The stress-tolerant Pokkali and stress-sensitive IR64 rice genotypes were subjected to 200 mM NaCl treatment for 2 h, and proteome profile was elucidated using the isobaric Tags for Relative and Absolute Quantitation (iTRAQ) approach (Fig. 1). iTRAQ which is a non-gel-based quantitative proteomics technique overcomes some of the drawbacks otherwise observed with 2-DE (Zieske 2006) and can be used to relatively quantify peptides at a global level (Ghosh et al. 2013). Proteome profiles of shoot and root tissues of both genotypes were studied separately to dissect primary and secondary signals generated by plants upon sensing salt stress. Roots serve as the primary site for salinity perception which then communicates these signals to shoots, the energy source of the developing seedlings. We specifically selected 2 h duration of stress treatment since we believe that it is the initial crucial phase of stress response which sets the foundation for stress adaptation in selected genotypes. Gaining an understanding of early triggering response is essential to unravel the basis of stress tolerance in plants. Our findings suggested that Pokkali proteome has an abundance of stress-responsive proteins under non-stress conditions especially the photosynthesis-related proteins. Importantly, these proteins exhibit higher expression in Pokkali than IR64 even under stress thereby contributing towards better adaptation of the latter towards stress.

## Results

### Determination of physiological perturbations in Pokkali and IR64 seedlings in response to salinity stress

**Fig. 2:**
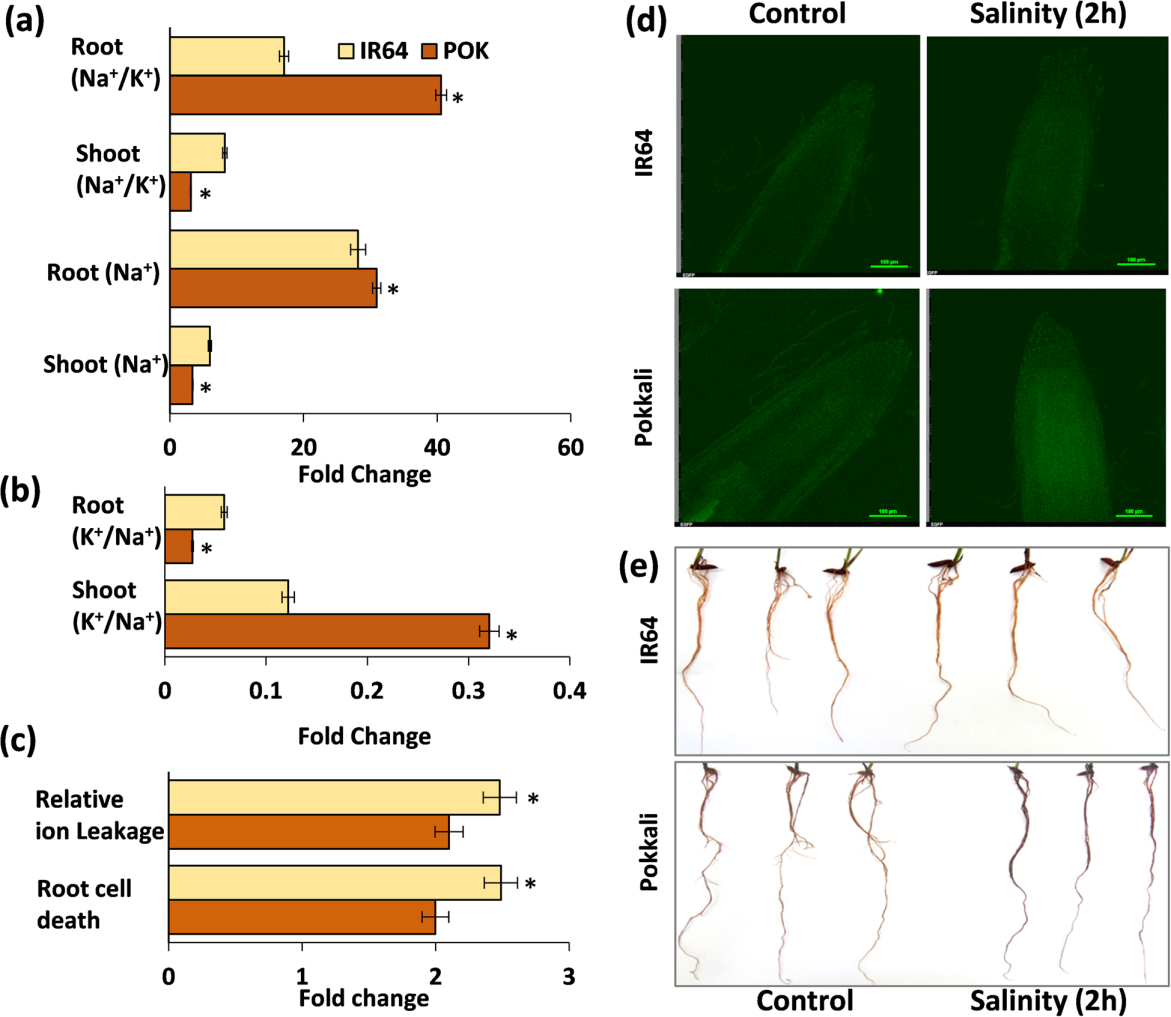
Determination of physiological parameters in Pokkali and IR64 rice seedlings in response to salinity stress. Ten-day old rice seedlings were subjected to salinity stress (200 mM NaCl) over a period of 2 h and changes were observed in shoot and root (**a**) Na^+^ and Na^+^/K^+^ ratio and **b** K^+^/Na^+^ ratio. **c** Determination of relative ion leakage (%) in shoot tissues under salinity stress and root cell death. **d** CoroNa dye staining of Pokkali and IR64 roots (**e**) DAB staining in roots under control and salinity stress (2 h) conditions. Significant differences as determined by student's t-test (*p*-values <0.05) are represented by an asterisk (*)

Ten-day old Pokkali and IR64 seedlings were treated for 2 h with 200 mM NaCl, and Na^+^ accumulation was measured in the shoot and root tissues. Under control conditions, Na^+^ levels were similar in both the genotypes (both roots and shoots). However, after 2 h of salinity treatment, Na^+^ levels increased by 6-folds (950.8 to 5674.3 ppm) and by 3.38-folds (777.6 to 2629.6 ppm) in shoots of IR64 and Pokkali respectively as compared to their untreated controls. Overall, sodium accumulation was 2.2-fold higher in IR64 shoots (5674.3 ppm) in comparison to Pokkali (2629.6 ppm) after 2 h of stress (Fig. 2a). By contrast, whereas Na^+^ levels increased by 28-folds in roots of IR64 seedlings (2038.41 to 57,426.71 ppm) after 2 h of the NaCl treatment, Pokkali roots exhibited a much higher increase (~ 31-folds) in Na^+^ levels (1919.71 to 59,426.71 ppm) under similar conditions (Fig. 2a). Change in Na^+^/K^+^ ratio was found to be 2.63-fold higher in IR64 shoots but 2.4-fold lower in roots of IR64 seedlings as compared to corresponding tissues of Pokkali (Fig. 2a). Further, change in K^+^/Na^+^ ratio was found to be 2.63-fold higher in Pokkali shoots but 2.15-fold lower in roots of Pokkali seedlings as compared to corresponding tissues of IR64 (Fig. 2b). However, percent change in electrolyte leakage (relative ion leakage) was ~ 1.5-fold higher in IR64 seedlings then Pokkali after 2 h of salinity stress (Fig. 2c). Further, CoroNa dye staining under control and stress conditions indicated higher Na accumulation in Pokkali roots at 2 h of stress in comparison to IR64 (Fig. 2d). We also measured root cell death and found that the uptake of the Evan blue dye (indication of dead cells) was more in IR64 than Pokkali (Additional file 1: Figure S1). Specifically, the root cell death rate was 1.5-fold pronounced in IR64 then Pokkali under salinity stress (Fig. 2c).

### Determination of reactive oxygen species (ROS) levels in response to salinity stress

Since stress is almost always accompanied by the production of reactive oxygen species (ROS), we checked the levels of H_2_O_2_ in both IR64 and Pokkali roots using DAB (3,3-diaminobenzidine) stain which indicates the levels of H_2_O_2_ through development of a dark brown color. Interestingly, we observed a higher DAB staining in Pokkali roots as compared to IR64 indicating higher H_2_O_2_ levels in Pokkali in response to salinity stress (Fig. 2e and Additional file 2: Figure S2). Taken together the present study reveals that within 2 h of salinity stress enough Na^+^ is build up in tissues which are affecting in turn, differentially affects the various physiological parameters between IR64 and Pokkali.

### iTRAQ based proteome analysis in seedlings of Pokkali and IR64 under control and salinity stress conditions

**Fig. 3:**
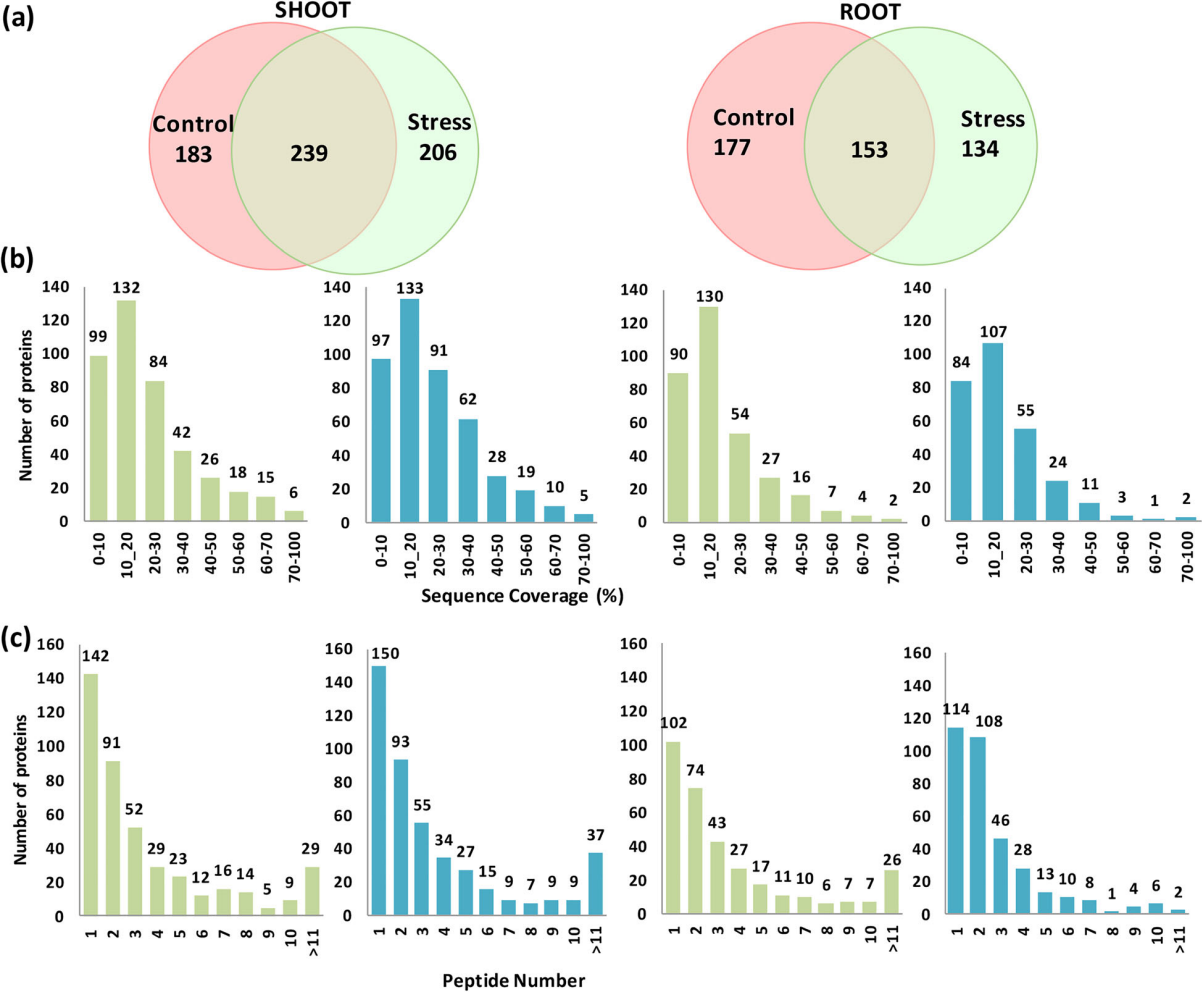
Summary of the proteomics data as revealed through iTRAQ. **a** Venn diagram showing the number of differentially accumulated proteins in shoot and root tissues of Pokkali viz. IR64 under control and salinity conditions. A total of 422 and 445 proteins were, detected under control and stress condition respectively in shoot tissues. Similarly, 330 and 287 proteins were detected under control and stress condition in root tissues, respectively. **b** Determination of peptide sequence coverage of the identified proteins. Graph shows the number of proteins distributed in different range of peptide sequence coverage. **c** Graphical representation of the distribution of the number of peptides for the identified proteins. Identified proteins were grouped based on the number of matched peptides

Our previous study on comparative temporal proteomics of Pokkali and IR64 rice seedlings has revealed some important physiological and proteome changes occurring in Pokkali under stress conditions (Lakra et al. 2018). However, in this work, changes in Pokkali and IR64 proteomes at early duration of stress were not investigated in detail and also changes in proteomes of two genotypes under non-stress conditions were not explored. Hence, to elucidate variations in the proteome of Pokkali and IR64 under control conditions as well as under stress, we carried out an iTRAQ-based proteome analysis and determined the proteins being differentially expressed in Pokkali in comparison to IR64 under normal and stress conditions. The proteins were detected at greater than 95% confidence with ProtScore cut off > 1.3. A total of 422 proteins under non-stress and 445 proteins under stress conditions were detected in the shoot tissues of Pokkali. 183 proteins were only detected under control conditions, 206 under only salt stress and 239 under both the conditions (Fig. 3a). Whereas in roots, of the 330 proteins detected under control (non-stress) conditions and 287 proteins detected under salinity stress, 153 were common to both (Fig. 3a). Further, peptide sequence coverage which indicates the percentage of the protein sequence covered by the identified peptides, was determined for the proteins. Maximum number of proteins showed 10–40% sequence coverage (Fig. 3b). Further, around 100–150 proteins in each sample were identified using only a single peptide. About 74–108 proteins had two peptide hits corresponding to the identified proteins and only 2–37 proteins had more than 11 identified peptides (Fig. 3c).

The differentially expressed proteins obtained from iTRAQ analysis were then quantitatively analyzed using appropriate selection criteria. The *p*-value threshold was kept 0.05. Fold change of greater than one in protein expression of Pokkali vs IR64 proteins was considered to be upregulated and less than one was taken to be downregulated. Based on these filters, we could find 86 proteins to be differentially expressed in shoots of Pokkali seedlings under non-stress conditions whereas under stress, 63 proteins were differentially expressed in Pokkali shoots in comparison to IR64 (Table 1, a-d). In roots, however lower number of proteins were obtained. Forty proteins under non-stress and eight proteins under stress were differentially expressed in Pokkali roots (Table 2, a-d). Interestingly, none of these identified root proteins were common between control and stressed proteomes of Pokkali. The differentially expressed proteins in both root and shoot tissues of Pokkali under control and salinity stress conditions have been listed in Tables 1 and 2.

**Table 1:** Differentially expressed proteins in shoot tissues of pokkali w.r.t IR64

Protein identity	% coverage	Peptides	POK/IR64	pvalue	MSU ID	Protein function
(A) Control UP						
Oxygen-evolving enhancer protein 1, chloroplast	70	60	8.5507	0.0022	LOC_Os01g31690.1	PS.lightreaction.photosystem II.PSII polypeptide subunits’
PsbP	73.6	48	6.0813	0.01	LOC_Os07g04840.1	PS.lightreaction.photosystem II.PSII polypeptide subunits’
Inorganic pyrophosphatase, putative	61.5	26	2.421	0.0041	LOC_Os02g52940.2	‘nucleotide metabolism.phosphotransfer and pyrophosphatases.misc’
Salt stress root protein RS1	76.5	19	6.6069	0.0013	LOC_Os01g13210.2	‘stress.abiotic.drought/salt’
Expressed protein	71.5	13	1.4588	0.0226	LOC_Os10g18340.2	‘not assigned.unknown’
Thioredoxin, putative	41.3	22	6.1376	0.0349	LOC_Os12g08730.1	‘redox.thioredoxin’
Superoxide dismutase [Cu-Zn]	62.6	19	99.0832	0.0032	LOC_Os08g44770.1	‘redox.dismutases and catalases’
Peptidyl-prolyl cis-trans isomerase	46.4	10	5.1051	0.0327	LOC_Os05g01270.1	‘cell.cycle.peptidylprolyl isomerase’
Thylakoid lumenal protein, putative	54.7	8	1.3932	0.0174	LOC_Os10g35810.1	biological process
NAD dependent epimerase/dehydratase	45.9	7	16.4437	0.0008	LOC_Os05g01970.5	‘protein.degradation’
ATP-dependent Clp protease ATP-binding subunit	14.5	6	3.8019	0.0046	LOC_Os04g32560.1	‘protein.degradation.serine protease’
Peptidyl-prolyl cis-trans isomerase	31.7	5	20.3236	0.0225	LOC_Os06g45340.1	‘protein.folding’
RAD23 DNA repair protein, putative	24.9	4	99.0832	0.0265	LOC_Os06g15360.1	‘DNA.repair’
Thylakoid lumenal 16.5 kDa protein	19.3	5	1.0666	0.0043	LOC_Os06g49160.1	Biological process
Calvin cycle protein CP12, putative,	44.4	5	7.8705	0.0334	LOC_Os01g19740.1	‘PS.calvin cycle’
Elongation factor protein	38.9	8	10.2802	0.0371	LOC_Os07g42300.1	‘protein.synthesis.elongation’
70 kDa heat shock protein	31.1	14	2.5351	0.0029	LOC_Os12g14070.1	‘stress.abiotic.heat’
Remorin, putative, expressed	38.4	3	1.6596	0.0182	LOC_Os04g45070.1	‘RNA.regulation of transcription.putative transcription regulator’
Proteasome subunit alpha type	31.7	3	3.0479	0.0444	LOC_Os11g40140.1	‘protein.degradation.ubiquitin.proteasom’
Oryzain alpha	10.3	4	1.4588	0.0025	LOC_Os04g55650.1	‘protein.degradation.cysteine protease’
acyl CoA binding protein, putative	79.1	5	1.1588	0.0204	LOC_Os06g02490.1	‘lipid metabolism.FA synthesis and FA elongation.acyl-CoA binding protein’
Lipid transfer protein-like	14.5	3	13.9316	0.033	LOC_Os08g42040.1	‘lipid metabolism.lipid transfer proteins etc’
Uridylyltransferase-related	23.9	3	1.2942	0.0067	LOC_Os08g14440.2	‘amino acid metabolism’
Nucleoside diphosphate kinase 1	34.2	2	99.0832	0.0265	LOC_Os07g30970.1	‘nucleotide metabolism.phosphotransfer and pyrophosphatases.nucleoside diphosphate kinase’
DnaK family protein	16.5	3	3.767	0.0049	LOC_Os02g53420.1	‘stress.abiotic.heat’
Hydroxyacylglutathione hydrolase	14.3	2	99.0832	0.019	OC_Os03g21460.1	‘Biodegradation of Xenobiotics.hydroxyacylglutathione hydrolase’
H0801D08.11 protein	14.3	2	87.9023	0.0185	LOC_Os04g58240.1	transport
Kinase, pfkB family,	22	2	2.884	0.0377	LOC_Os08g02120.1	‘major CHO metabolism.degradation.sucrose.fructokinase’
Histone H2B	16.5	2	1.1803	0.0033	LOC_Os05g49860.1	‘DNA.synthesis/chromatin structure.histone’
Putative group 3 LEA protein	32	2	1.7701	0.0191	LOC_Os05g46480.1	reproduction, post-embryonic development, embryo development
Putative Ras-GTPase-activating protein binding protein 1	6.5	2	87.0964	0.0182	LOC_Os02g29480.1	‘protein.targeting.nucleus’
Coproporphyrinogen III oxidase, chloroplast	16.8	1	87.9023	0.0189	LOC_Os04g52130.1	‘tetrapyrrole synthesis.coproporphyrinogen III oxidase’
OsAPx6 - Stromal Ascorbate Peroxidase	25.6	5	8.091	0.0366	LOC_Os12g07820.1	‘redox.ascorbate and glutathione.ascorbate’
Peptidyl-prolyl cis-trans isomerase	26.8	1	99.0832	0.0192	LOC_Os02g52290.1	‘protein.folding’
Methionyl-tRNA synthetase, putative	23	1	87.9023	0.0185	LOC_Os04g23820.1	‘protein.aa activation’
Dihydroorotate dihydrogenase protein	9.4	1	1.0965	0.027	LOC_Os02g50350.1	‘nucleotide metabolism.degradation.pyrimidine.dihydrouracil dehydrogenase’
Thylakoid lumenal protein	24.6	4	1.1482	0.0409	LOC_Os02g42960.1	Biological process
cDNA clone:001–039-F07	24.5	3	2.9648	0.0454	LOC_Os12g02370.2	‘secondary metabolism.flavonoids.chalcones’
Putative uncharacterized protein	23.8	3	31.0456	0.0009	LOC_Os01g34700.1	biological_process/metabolic process
Non-specific lipid-transfer protein	27.1	3	64.8634	0.0186	LOC_Os11g02369.1	LTPL7 - Protease inhibitor/seed storage/LTP family protein precursor
EF hand family protein	27.9	3	2.0137	0.0175	LOC_Os03g29770.1	‘signalling.calcium’
Os05g0291700 protein	25.8	2	2.2491	0.0411	LOC_Os05g22614.4	metabolic process/biological process
ATP synthase subunit alpha	15.3	2	1.4997	0.0367	LOC_Os09g08910.1	‘mitochondrial electron transport / ATP synthesis.F1-ATPase’
High mobility group protein	17.8	2	99.0832	0.0172	LOC_Os06g51220.4	cellular component organization
stress responsive protein	8.3	2	1.2474	0.0481	LOC_Os03g21040.2	Biological process
phytocyanin-related protein Pn14	10.4	2	87.9023	0.0188	LOC_Os08g17160.1	‘misc.plastocyanin-like’
Putative uncharacterized protein/aminotransferase	3.9	2	87.9023	0.0182	LOC_Os04g52440.1	‘amino acid metabolism.synthesis.central amino acid metabolism.GABA.GABA transaminase’
PREDICTED: DNA-binding protein MNB1B	43.4	3	8.0168	0.0009	LOC_Os02g26440.1	protein metabolic process
xyloglucan endotransglycosylase/hydrolase protein 8	15.9	2	6.5464	0.0388	LOC_Os08g13920.1	‘cell wall.modification’
ribosomal protein/LOC_Os02g33140.1	20.2	1	99.0832	0.0188	LOC_Os02g33140.1	‘protein.synthesis.ribosomal protein.eukaryotic.40S subunit.S14’
Putative Photosystem I reaction center subunit IV	12.8	1	8.0168	0.0191	LOC_Os07g25430.1	‘PS.lightreaction.photosystem I.PSI polypeptide subunits’
Actin-1	15.9	1	99.0832	0.019	LOC_Os03g50885.1	cellular component organization
Putative peptidyl-prolycis-trans isomerase protein	6.7	1	1.6904	0.0385	LOC_Os07g37830.1	‘cell.cycle.peptidylprolyl isomerase’
HMG protein	31.8	1	87.9023	0.0179	LOC_Os04g47690.2	Biological process (DNA binding)
Putative uncharacterized protein	5.3	1	9.3756	0.0384	LOC_Os12g15470.2	‘protein.degradation.serine protease’
ferredoxin-dependent glutamate synthase,	2.1	1	64.2688	0.0187	LOC_Os07g46460.1	‘N-metabolism.ammonia metabolism.glutamate synthase’
Os07g0585000 protein	8.1	1	87.9023	0.0183	LOC_Os07g39620.2	‘stress.abiotic.cold’
putative CR9/Light-regulated protein	22.7	1	99.0832	0.0182	LOC_Os01g01340.1	vacuolar transport
GDP-mannose 3,5-epimerase 1	5.6	1	99.0832	0.0187	LOC_Os10g28200.1	‘redox.ascorbate and glutathione.ascorbate.GME’
Lipid transfer protein-like	4.8	1	99.0832	0.0181	LOC_Os07g09970.1	‘misc.protease inhibitor/seed storage/lipid transfer protein (LTP) family protein’
TA9 protein-like	1.5	1	99.0832	0.0181	LOC_Os01g47430.2	DUF1296 domain containing protein
Os01g0763650 protein	7.9	1	99.0832	0.0188	LOC_Os01g0763700	lipid metabolic process
dirigent protein 22	6.3	1	99.0832	0.0189	LOC_Os11g07670.1	‘stress.biotic.PR-proteins’
KE2 family protein	5.4	1	99.0832	0.0186	LOC_Os12g30060.2	protein metabolic process
Glucan endo-1,3-beta-glucosidase 5	5.5	1	99.0832	0.0198	LOC_Os11g36940.1	‘misc.beta 1,3 glucan hydrolases.glucan endo-1,3-beta-glucosidase’
Os08g0459300 protein	5.8	1	85.5067	0.019	LOC_Os08g35710.1	‘PS.lightreaction.other electron carrier (ox/red).ferredoxin’
LOC_Os03g45340.1/hsp20/alpha crystallin family protein	15.6	1	25.3513	0.019	LOC_Os03g45340.1	response to abiotic stimulus
Os08g0530200/Putative ribosomal protein L17	15.8	2	6.3096	0.0441	LOC_Os08g41810.1	‘protein.synthesis.ribosomal protein.eukaryotic.60S subunit.L17’
HIPL1 protein, putative	6.1	1	15.1356	0.0188	LOC_Os12g44230.1	‘cell.organisation’
(B) Control Down						
T-complex protein, putative,	48.3	29	0.1585	0	LOC_Os12g17910.1	‘protein.folding’
ATP synthase subunit beta	46.4	18	0.4786	0.026	LOC_Os10g21266.1	‘mitochondrial electron transport / ATP synthesis.F1-ATPase’
Putative transketolase	28	10	0.0692	0.0002	LOC_Os06g04270.1	‘PS.calvin cycle.transketolase’
Dehydroascorbate reductase	65.3	9	0.0929	0.0485	LOC_Os05g02530.1	‘redox.ascorbate and glutathione.ascorbate’
Peptidyl-prolyl cis-trans isomerase	61.1	10	0.092	0.0103	LOC_Os02g02890.1	‘cell.cycle.peptidylprolyl isomerase’
Glyoxalase	26.5	9	0.1472	0.0436	LOC_Os08g09250.2	amino acid metabolism.degradation.aspartate family.threonine’
peroxiredoxin, putative	35.6	4	0.038	0.0465	LOC_Os02g09940.1	‘redox.peroxiredoxin’
T-complex protein, putative	14.3	4	0.3373	0.0418	LOC_Os06g02380.2	‘protein.folding’
Carbonic anhydrase	32.7	5	0.5395	0.0208	LOC_Os01g45274.1	‘TCA / org. Transformation.carbonic anhydrases’
glycine-rich protein 2, putative	47.2	3	0.138	0.014	LOC_Os01g36570.1	‘stress.abiotic.cold’
plasminogen activator inhibitor 1 RNA-binding protein	30.5	4	0.5445	0.0414	LOC_Os05g45660.2	‘RNA.RNA binding’
NAD dependent epimerase/dehydratase	8.4	1	0.278	0.0448	LOC_Os07g11110.1	‘RNA.regulation of transcription.unclassified’
60 kDa chaperonin alpha subunit	39.8	17	0.0871	0.0445	LOC_Os11g47970.1	AAA-type ATPase family protein
Ribulose bisphosphate carboxylase small chain	57.7	14	0.1169	0.0039	LOC_Os12g17600.1	ribulose bisphosphate carboxylase small chain
Putative uncharacterized protein	34.9	3	0.1614	0.0324	LOC_Os03g08800.1	CutA, chloroplast precursor, putative
Protein CutA, chloroplast,	20.4	1	0.9204	0.043	LOC_Os04g50110.1	RNA recognition motif containing protein,
Putative uncharacterized protein	8.3	2	0.0111	0.0444	LOC_Os03g20630.2	histidine triad family protein,
(C) Stress UP						
Oxygen-evolving enhancer protein 1, chloroplast	58	43	15.7036	0.0028	LOC_Os01g31690.1	PS.lightreaction.photosystem II.PSII polypeptide subunits’
Ribulose bisphosphate carboxylase large chain 1	53.5	41	5.4954	0.0126	LOC_Os10g21268.1	‘PS.calvin cycle.rubisco large subunit’
PsbP	71.3	32	19.0546	0.0129	LOC_Os07g04840.1	PS.lightreaction.photosystem II.PSII polypeptide subunits’
Putative inorganic pyrophosphatase	65.4	22	4.7424	0.0126	LOC_Os02g52940.2	‘nucleotide metabolism.phosphotransfer and pyrophosphatases.misc’
Catalase	36.2	16	3.2509	0.0041	LOC_Os02g02400.1	‘redox.dismutases and catalases’
protein|ribosome recycling factor, putative	49.6	18	3.1623	0.0001	LOC_Os07g38300.1	‘cell.division’
Phosphoribulokinase	45.2	16	2.5119	0.0214	LOC_Os02g47020.1	‘PS.calvin cycle.PRK’
protein|salt stress root protein RS1	71.6	13	7.5858	0.0005	LOC_Os01g13210.2	‘stress.abiotic.drought/salt’
Carbonic anhydrase	53.3	16	8.1658	0.0016	LOC_Os01g45274.1	‘TCA / org. Transformation.carbonic anhydrases’
RNA recognition motif containing protein	49	12	5.4954	0.002	LOC_Os09g10760.1	‘RNA.RNA binding’
ABA/WDS induced protein,	58	10	4.8306	0.0302	LOC_Os11g06720.1	abscisic stress-ripening
protein|expressed protein	60.6	8	1.9409	0.0023	LOC_Os10g18340.2	‘not assigned.unknown’
30S ribosomal protein S1, chloroplast	23.7	6	2.0893	0.0321	LOC_Os03g20100.1	‘protein.synthesis.ribosomal protein.prokaryotic.unknown organellar.30S subunit.S1’
30S ribosomal protein S31, chloroplast precursor,	37.3	5	3.1046	0.001	LOC_Os05g09400.3	translation
Putative group 3 LEA protein	31.5	3	5.445	0.001	LOC_Os05g46480.1	reproduction, post-embryonic development, embryo development
Ankyrin repeat domain protein 2,	15.1	3	1.5136	0.0012	LOC_Os03g63480.1	‘RNA.regulation of transcription.AtSR Transcription Factor family’
protein|OsCML7 - Calmodulin-related calcium sensor protein	32.4	3	1.6596	0.0248	LOC_Os08g02420.1	‘signalling.calcium’
Class III peroxidase 125	13.1	3	6.2517	0.0012	LOC_Os10g02040.1	‘misc.peroxidases’
linker histone H1 and H5 family protein	20.2	1	52.9663	0.0181	LOC_Os08g33190.1	‘DNA.synthesis/chromatin structure.histone’
Lipid transfer protein-like	9.5	2	83.1764	0.0179	LOC_Os08g42040.1	‘lipid metabolism.lipid transfer proteins etc’
1,2-dihydroxy-3-keto-5-methylthiopentene dioxygenase protein	26.3	1	1.028	0.0176	LOC_Os03g06620.1	‘metal handling.regulation’
oxygen evolving enhancer protein 3	66.8	47	11.1686	0.01	LOC_Os07g36080.1	‘PS.lightreaction.photosystem II.PSII polypeptide subunits’
Ribulose bisphosphate carboxylase/oxygenase activase,	57.2	38	3.4995	0.0003	LOC_Os11g47970.1	‘PS.calvin cycle.rubisco interacting’
ferredoxin-dependent glutamate synthase,	13	9	2.6546	0.0018	LOC_Os07g46460.1	‘N-metabolism.ammonia metabolism.glutamate synthase’
Putative uncharacterized protein	40.5	9	9.3756	0.0202	XP_015634836.1	probable peroxisomal membrane protein PEX13
Os01g0326000 protein/peroxidase 1	19.9	5	2.0137	0.0473	LOC_Os01g22230.1	‘misc.peroxidases’
CDGSH iron-sulfur domain-containing protein NEET	52.3	4	10.3753	0.0159	LOC_Os07g28400.1	biological_process
Os04g0445200 protein	56.5	3	3.4995	0.0045	LOC_Os04g36760.1	NO
Q8LQN2|Q8LQN2_ORYSJ	24.6	5	1.3932	0.0463	LOC_Os01g57570.1	NADPH-dependent FMN reductase domain containing protein
BBTI7 - Bowman-Birk type bran trypsin inhibitor precursor	15.1	2	2.2491	0.0023	LOC_Os01g03390.1	BBTI7 - Bowman-Birk type bran trypsin inhibitor precursor
OSJNBa0086O06.22 protein/31 kDa ribonucleoprotein	10.7	3	1.9588	0.0013	LOC_Os04g50110.1	‘RNA.RNA binding’
Os06g0671900 protein/Q0DA75|Q0DA75_ORYSJ	13.6	2	1.2246	0.0378	LOC_Os06g46000.1	tubulin/FtsZ domain containing protein
LTPL52 - Protease inhibitor/seed storage	21.8	2	2.3768	0.0009	LOC_Os03g26820.1	transport
Putative SHOOT1 protein	18.8	1	5.2966	0.0319	LOC_Os07g07540.1	response to stress
Glutamate dehydrogenase	7.8	1	99.0832	0.019	LOC_Os03g58040.2	‘N-metabolism.N-degradation.glutamate dehydrogenase’
Alpha-tubulin	9.1	1	42.0727	0.0184	LOC_Os11g14220.1	cell.organisation’/response to abiotic stimulus
(D) Stress Down						
enolase, putative	37.1	9	0.4742	0.0065	LOC_Os10g08550.1	‘glycolysis.cytosolic branch.enolase’
ubiquitin fusion protein, putative	61.2	13	0.3311	0.0313	LOC_Os09g39500.1	‘protein.degradation.ubiquitin.ubiquitin’
Glyoxalase	33.7	10	0.2754	0.0121	LOC_Os08g09250.2	amino acid metabolism.degradation.aspartate family.threonine’
Glyceraldehyde-3-phosphate dehydrogenase	37.1	10	0.3221	0.0005	LOC_Os04g40950.1	‘glycolysis.cytosolic branch.glyceraldehyde 3-phosphate dehydrogenase (GAP-DH)’
Malate dehydrogenase	37.4	7	0.3048	0.0326	LOC_Os10g33800.1	‘TCA / org. Transformation.other organic acid transformaitons.cyt MDH’
Nucleoside diphosphate kinase	48.3	5	0.2051	0.0283	LOC_Os10g41410.2	‘nucleotide metabolism.phosphotransfer and pyrophosphatases.nucleoside diphosphate kinase’
Stress responsive protein	20.3	3	0.1644	0.0276	LOC_Os01g01450.1	Stress responsive protein
L11 domain containing ribosomal protein	31.3	3	0.0316	0.0081	LOC_Os02g47140.1	‘protein.synthesis.ribosomal protein.eukaryotic.60S subunit.L12’
Class III peroxidase 65	21.6	6	0.1905	0.0181	LOC_Os05g04380.1	‘misc.peroxidases’
RNase S-like protein	30.2	3	0.0119	0.0067	LOC_Os09g36680.1	‘RNA.processing.ribonucleases’
Phenylalanine ammonia-lyase	13	3	0.7727	0.0162	LOC_Os02g41630.2	‘secondary metabolism.phenylpropanoids.lignin biosynthesis.PAL’
Probable aldo-keto reductase 2	19.1	2	0.0938	0.016	LOC_Os04g26910.1	‘hormone metabolism.auxin.induced-regulated-responsive-activated’
ranBP1 domain containing protein	5.7	2	0.0316	0.0075	LOC_Os05g28190.1	‘signalling.G-proteins’
Putative Ras-GTPase-activating protein binding protein 1	13.1	1	0.2014	0.0451	LOC_Os02g29480.1	‘protein.targeting.nucleus’
Fructose-bisphosphate aldolase	30.2	8	0.0296	0.0041	LOC_Os01g67860.1	‘PS.calvin cycle.aldolase’
enoyl-acyl-carrier-protein reductase NADH	22.6	5	0.6982	0.0133	LOC_Os08g23810.1	‘lipid metabolism.FA synthesis and FA elongation.enoyl ACP reductase’
dehydrogenase, putative	27.2	3	0.912	0.0169	LOC_Os08g29170.1	‘misc.oxidases - copper, flavone etc.’
SOR/SNZ family protein	22.7	4	0.6138	0.0257	LOC_Os10g01080.1	‘Co-factor and vitamine metabolism’
ketol-acid reductoisomerase	10.3	3	0.9376	0.0359	LOC_Os05g49800.1	‘amino acid metabolism.synthesis.branched chain group.common’
NAD dependent epimerase/dehydratase family	10.6	3	0.0283	0.0056	LOC_Os03g16980.1	‘cell wall.precursor synthesis.UXS’
erythronate-4-phosphate dehydrogenase domain containing	22.6	3	0.053	0.0119	LOC_Os06g29180.1	‘C1-metabolism’
transaldolase	12.3	2	0.1019	0.0146	LOC_Os01g70170.1	‘OPP.non-reductive PP.transaldolase’
peptidase, T1 family	11.5	2	0.0313	0.04	LOC_Os02g42320.2	‘protein.degradation.ubiquitin.proteasom’
actin, putative	21	2	0.4966	0.048	LOC_Os11g06390.4	‘cell.organisation’
RNA recognition motif containing protein	41.3	3	0.5058	0.0222	LOC_Os01g68790.2	‘RNA.RNA binding’
osmotin, putative	11.6	2	0.5012	0.0451	LOC_Os12g38170.1	‘stress.abiotic’
NADPH-dependent FMN reductase domain containing protein	19.2	3	0.0121	0.0492	LOC_Os08g04460.1	‘lipid metabolism.“exotics” (steroids, squalene etc)’

**Table 2:** Differentially expressed proteins in root tissues of pokkali w.r.t IR64

Protein identity	% coverage	Peptides	POK/IR64	pvalue	MSU ID	Protein function
(A) Control UP						
Os10g0191300 protein	62.5	43	2.208	0.0038	LOC_Os10g11500.1	‘stress.biotic’
Phenylalanine ammonia-lyase	37.4	32	1.5417	0.012	LOC_Os02g41630.2	‘secondary metabolism.phenylpropanoids.lignin biosynthesis.PAL’
Class III peroxidase 86	48.2	21	1.977	0.0032	LOC_Os06g35520.1	‘misc.peroxidases’
Putative r40c1 protein-rice	41.6	14	1.8707	0.0498	LOC_Os03g21040.2	Stress responsive protein
Os03g0712700 protein	28.2	10	2.5823	0.0167	LOC_Os03g50480.1	‘not assigned.unknown’
Malate dehydrogenase	41.6	10	17.2187	0.0011	LOC_Os10g33800.1	‘TCA / org. Transformation.other organic acid transformaitons.cyt MDH’
Methylmalonate semi-aldehyde dehydrogenase	23.6	11	2.0137	0.0305	LOC_Os07g09060.1	‘amino acid metabolism.degradation.branched-chain group.valine’
UTP--glucose-1-phosphate uridylyltransferase, putative	30.5	10	5.5976	0.0071	LOC_Os09g38030.1	biological process
Beta-1,3-glucanase (Fragment)	30.4	10	2.2909	0.0103	LOC_Os01g51570.1	‘misc.beta 1,3 glucan hydrolases.glucan endo-1,3-beta-glucosidase’
peroxidase precursor, putative,	24	7	5.5976	0.0038	LOC_Os05g06970.1	‘misc.peroxidases’
|inhibitor I family protein, putative	76.1	5	1.0471	0.0056	LOC_Os01g42860.1	‘not assigned.unknown’
|leucine aminopeptidase, chloroplast precursor	10	4	3.6644	0.0376	LOC_Os02g55140.1	protein.degradation’
6-phosphogluconate dehydrogenase, decarboxylating	20.4	4	2.1086	0.0291	LOC_Os06g02144.1	‘OPP.oxidative PP.6-phosphogluconate dehydrogenase’
succinyl-CoA ligase beta-chain, mitochondrial precursor	14.7	3	3.0761	0.0114	LOC_Os02g40830.1	‘TCA / org. Transformation.TCA.succinyl-CoA ligase’
Os02g0582900 protein	37.2	8	16.293	0.0177	LOC_Os02g0582900	NOT
Adenine phosphoribosyltransferase 1, putative,	22.1	3	99.0832	0.0004	LOC_Os12g39860.1	‘nucleotide metabolism.salvage.phosphoribosyltransferases.aprt’
Superoxide dismutase			3.0479	0.0509	LOC_Os05g25850.1	redox.dismutases and catalases’
Putative isomerase	15.3	2	22.9087	0.0085	LOC_Os03g61330.2	‘amino acid metabolism.degradation.aromatic aa.tyrosine’
Proteasome subunit alpha type	22.4	2	1.888	0.031	LOC_Os11g40140.1	‘protein.degradation.ubiquitin.proteasom’
Sucrose synthase 2	4.5	1	2.3768	0.0195	LOC_Os06g09450.3	‘major CHO metabolism.degradation.sucrose.Susy’
60 kDa chaperonin	9.2	1	71.1214	0.0188	LOC_Os10g32550.1	‘protein.folding’
Os02g0583700 protein/hypothetical protein	37.7	5	38.7258	0.0255	LOC_Os02g37250.1	‘not assigned.unknown’
Malic enzyme (Fragment) OS=*Oryza sativa subsp. japonica* GN=Os01g0723400 PE = 3 SV = 1	26.2	11	2.5351	0.0012	LOC_Os01g52500.5	‘TCA / org. Transformation.other organic acid transformaitons.malic’
Aldehyde dehydrogenase OS=Oryza sativa GN = Aldh PE = 2 SV = 1	20.8	7	3.4674	0.0314	LOC_Os06g15990.1	fermentation.aldehyde dehydrogenase’
Os02g0196800 protein OS=Oryza sativa subsp. japonica GN=OJ1524_D08.17 PE = 2 SV = 1	16.3	2	7.656	0.038	LOC_Os02g10310.1	‘amino acid metabolism.degradation.aromatic aa.tyrosine’
Putative chaperonin 21 OS=Oryza sativa subsp. japonica GN=B1172G12.2 PE = 3 SV = 1	30.1	2	1.6144	0.0382	LOC_Os06g09688.1	‘protein.folding’
Class III peroxidase 70 OS=Oryza sativa subsp. japonica GN = prx70 PE = 3 SV = 1	16.7	3	99.0832	0.0187	LOC_Os05g04490.1	‘misc.peroxidases’
endo-1,3;1,4-beta-D-glucanase precursor	15.5	1	8.2414	0.0397	LOC_Os05g33100.1	response to abiotic stimulus
Class III peroxidase 122 OS=Oryza sativa subsp. japonica GN=OJ1118_B06.10 PE = 3 SV = 1	24.2	1	8.9536	0.0364	LOC_Os09g29490.1	‘misc.peroxidases’
MPI, putative OS=Oryza sativa subsp. japonica GN = LOC_Os12g36220 PE = 4 SV = 2	28.8	2	87.9023	0.0168	LOC_Os12g36220.1	negative regulation of endopeptidase activity, response to stress
similar to oxygen evolving enhancer protein 3 domain containing protein, Ferredoxin-NADP reductase binding protein	12	1	54.9541	0.0184	LOC_Os07g36080.1	protein modification process
Putative L-asparaginase OS=Oryza sativa subsp. japonica GN=OSJNBa0087M10.11 PE = 4 SV = 1	7.1	1	99.0832	0.0175	LOC_Os03g40070.1	‘amino acid metabolism.degradation.aspartate family.asparagine.L-asparaginase’
(B) Control Down						
Acidic PR-1 type pathogenesis-related protein PR-1a OS=Oryza sativa subsp. japonica GN=PR-1a PE = 2 SV = 1	46.4	7	0.5702	0.0429	LOC_Os07g03710.1	‘stress.biotic’
Os08g0162800 protein OS=Oryza sativa subsp. japonica GN=P0577B11.140 PE = 4 SV = 1	65.9	9	0.2679	0.0035	LOC_Os08g06550.1	‘lipid metabolism.FA synthesis and FA elongation.acyl-CoA binding protein’
Ascorbate peroxidase OS=Oryza sativa subsp. japonica GN=Os07g0694700 PE = 2 SV = 1	37.1	5	0.4246	0.028	LOC_Os07g49400.1	‘redox.ascorbate and glutathione.ascorbate’
Triosephosphate isomerase OS=Oryza sativa subsp. japonica GN=P0569E11.2–1 PE = 3 SV = 1	16.8	4	0.0855	0.0233	LOC_Os09g36450.1	‘PS.calvin cycle.TPI’
peroxidase precursor,	26.5	7	0.1406	0.0115	LOC_Os04g59190.1	‘misc.peroxidases’
fructose-bisphospate aldolase isozyme	10.7	2	0.1127	0.0165	LOC_Os11g07020.1	carbohydrate metabolic process
Ubiquitin family domain containing protein	23.1	3	0.6252	0.0184	LOC_Os02g10510.1	‘protein.degradation.ubiquitin.ubiquitin’
RNA recognition motif containing protein	15.3	1	0.0111	0.0487	LOC_Os01g68790.2	‘RNA.RNA binding’
(C) Stress UP						
Os01g0326000/ peroxidase	26.3	6	23.1206	0.0005	LOC_Os01g22230.1	'misc.peroxidases'
triosephosphate isomerase cytosolic	14.5	2	2.3335	0.0502	LOC_Os01g05490.1	'PS.calvin cycle.TPI'
calreticulin precursor protein	25.7	6	4.1305	0.0682	LOC_Os07g14270.3	'signalling.calcium'
Os02g0167300/tubulin beta-5 chain	7.8	3	2.6792	0.0341	LOC_Os02g07060.1	'cell.organisation'
(D) Stress Down						
5-methyltetrahydropteroyltriglutamate-homocysteine methyltransferase, 5-metH	19.3	4	0.5248	0.0053	LOC_Os12g42876.1	'amino acid metabolism.synthesis.aspartate family.methionine'
BBTI4 - Bowman-Birk type bran trypsin inhibitor precursor	19.5	2	0.2938	0.0454	LOC_Os01g03340.1	NO
retrotransposon protein, putative, Ty1-copia subclas	31.6	2	0.3251	0.0044	LOC_Os08g03520.1	'stress.abiotic.cold'
thiol protease SEN102 precursor	19	4	0.4831	0.0067	LOC_Os09g39070.1	'protein.degradation.cysteine protease'

### Functional classification of differentially abundant proteins in seedlings of Pokkali under control and stress conditions

**Fig. 4:**
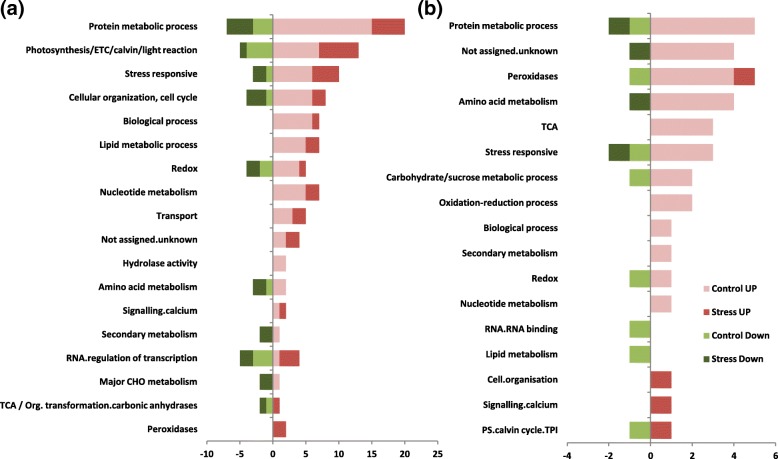
Functional classification of differentially regulated proteins in (**a**) shoot and (**b**) root tissues of Pokkali in comparison to IR64 under control and stress conditions. The classification is based on the biological processes obtained from the GO classification and MSU database. PsbP;PS.lightreaction.photosystem II, PS.calvin cycle.TPI (Triose phosphate isomerase), TCA; tricarboxylic acid

In order to analyze the differential response of Pokkali and IR64, differentially expressed proteins were classified into functional categories. Eighteen major categories of proteins were observed in the shoot proteome of Pokkali whereas root proteome comprised of 17 major functional categories (Fig. 4a, b). Under non-stress conditions, proteins belonging to the protein metabolic process-related and photosynthesis/ETC/Calvin/light reaction-related categories were found to be most enriched; comprising 22% and 13% of the total differential shoot proteome, respectively. The third major category was of stress-responsive proteins and those involved in cellular organization/cell cycle, both comprising 8% of the differential shoots proteome under control conditions. Interestingly, these categories remained enriched even after 2 h of stress (Fig. 4a).

Inspection of differential root proteome of Pokkali under non-stress conditions revealed an abundance of metabolic process-related proteins which formed 13% of the total differential proteome followed by 'function not assigned' category (Fig. 4b). The other major category being differentially regulated was found to be of peroxidase (11%) which was followed by category of stress-responsive and amino acid metabolism related proteins (8.5%). However, in the stressed root proteome of Pokkali, functional categories such as, peroxidase family proteins, PS.calvin cycle.TPI and signaling proteins were found to be selectively enriched (Fig. 4b).

### Proteins with very high abundance in Pokkali proteome under non-stress conditions

Considering the fact that the unstressed proteome of Pokkali had higher abundance of many proteins belonging to different categories in comparison to IR64, we specifically analyzed those proteins which differed markedly in their abundance in the two genotypes and thus, used > 60-fold change criteria for selection of those proteins in the differential proteome. Among the shoot proteins with > 60-fold change expression in Pokkali, are several abiotic and biotic stress responsive candidates such as glyoxalase II, superoxide dismutase, peptidyl-prolyl cis-trans isomerase and dirigent protein 22 (Additional file 3: Table S1). Further, even a photosynthetic protein, phytocyanin-related protein Pn14, was found to be highly expressed in Pokkali. Furthermore, a ferredoxin-dependent glutamate synthase protein showed 64-fold increase in Pokkali in comparison to IR64 under control conditions.

In contrast to shoot proteome, which had 27 proteins with greater than 60-fold expression, root proteome had only five such proteins, which included, adenine phosphoribosyltransferase 1, peroxidase, chaperonin and L-asparaginase (Additional file 3: Table S1). Peroxidases and chaperonins are known to be the key players in stress response. Thus, Pokkali proteome appeared to possess huge differences in abundance of some proteins involved in stress response and adaptation in comparison to IR64. However, it should be noted that there are other stress-responsive proteins in the Pokkali proteome as well, which though are more abundant than in IR64 but have not been considered here (present in higher amount with a little change in their abundance) and hence, need low increment in their abundance for appropriately performing their functions.

### Hierarchical clustering of differentially expressed proteins in Pokkali vs IR64

**Fig. 5:**
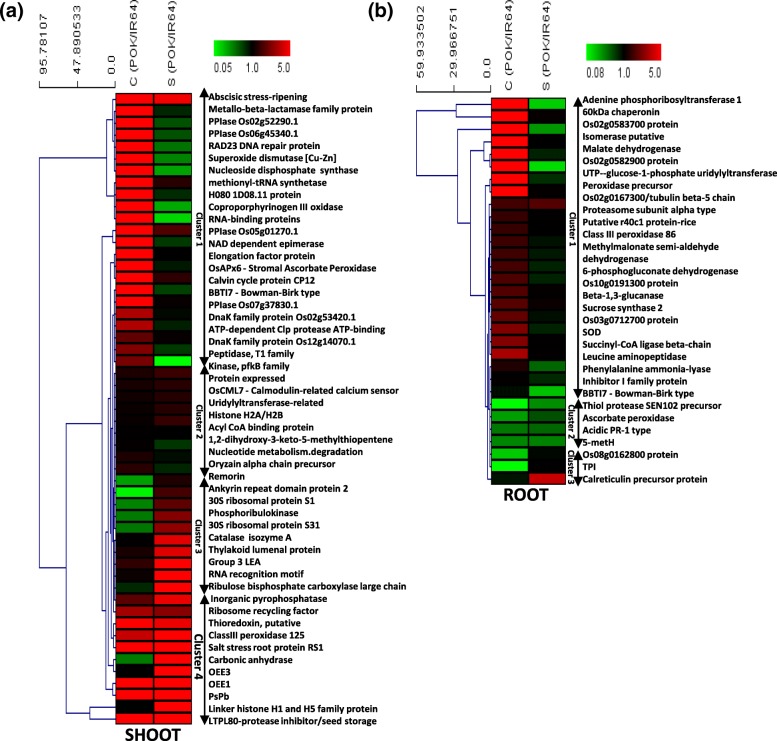
Hierarchical clustering of differentially expressed proteins obtained from the shoot (**a**) and root (**b**) tissues of Pokkali w.r.t IR64 under control and salinity stress conditions. Differentially expressed proteins were clustered using TMeV software and represented as heat maps

Hierarchical clustering analysis was performed to understand the pattern of protein expression in Pokkali vs IR64 under control and stress conditions. For this, proteins common between control and stressed proteome of Pokkali and IR64 were taken for analysis. Twenty three proteins grouped as cluster one, were found to be highly expressed in Pokkali under control conditions in comparison to IR64 but under salinity stress, their expression pattern reversed where these proteins exhibited higher expression levels in IR64 than Pokkali. These proteins included four prolyl peptidyl cis-trans isomerases (PPiases), a superoxide dismutase protein, a metallo-beta lactamase protein, DnaK protein, OsAPX6, coproporphyrinogen oxidase and Calvin cycle protein CP12 (Fig. 5a). However, in this cluster, there were few proteins which were though highly expressed under control conditions in Pokkali but little change under stress conditions such as peptidyl-prolyl cis-trans isomerases (Os02g52290.1 and Os06g45340.1), peptidyl-prolyl cis-trans isomerase FKBP type, elongation factor and DnaK family protein (Os12g14070.1 and Os02g53420.1) (Listed in Additional file 4: Table S2). Further, few proteins grouped in cluster 2, such as, OsCML7 and a histone protein, exhibited a minimal change in their expression pattern under stress when compared to the differential control proteome of Pokkali. The third type of cluster was composed of proteins which exhibited low expression in Pokkali viz. IR64 under non-stress conditions but showed a significant increase in Pokkali under stress. This category included various proteins such as, ankyrin repeat domain protein 2, catalase, 30S ribosomal proteins, thylakoid luminal protein, LEA protein, ribulose bisphosphate carboxylase and phosphoribulokinase (Cluster 3; Fig. 5a). Cluster 4, which comprised of proteins with increased expression in Pokkali with respect to IR64 under both control and stress conditions were also observed. This group comprised proteins such as oxygen evolving enhancer proteins (OEE), thioredoxin, Class III peroxidase, and lipid transfer proteins (Fig. 5a).

Similarly, the differentially expressed proteins in root proteome of Pokkali were also found to be clustered into various groups (Fig. 5b). A number of proteins like malate dehydrogenase, peroxidase, 60 kDa chaperonin protein, adenine phosphoribosyltransferase 1 (APRT1), methylmalonate semi-aldehyde (MMSDH), 6-phosphogluconate dehydrogenase (6PGDH), Succinyl-CoA ligase (SCoAL), and leucine aminopeptidase were found to be highly abundant under non-stress conditions in Pokkali then to IR64 but under stress, levels of these proteins either increased in IR64 with respect to Pokkali or decreased with respect to their control levels in Pokkali (Cluster 1; Fig. 5b). Another group comprised of proteins whose expression levels remained low in control as well as in stress conditions in Pokkali with respect to IR64 and included proteins such as, thiol protease, acidic PR-1 type pathogenesis related protein, and sucrose synthase (Cluster 2; Fig. 5b). The third group comprised of proteins like calreticulin and triose phosphate isomerase which were either low in expression or exhibited only marginally higher expression in Pokkali with respect to IR64 under control conditions but their expression ratio (Pokkali vs. IR64) increased under stress conditions (Cluster 3; Fig. 5b).

### Transcript abundance of selected genes using qRT-PCR

**Fig. 6:**
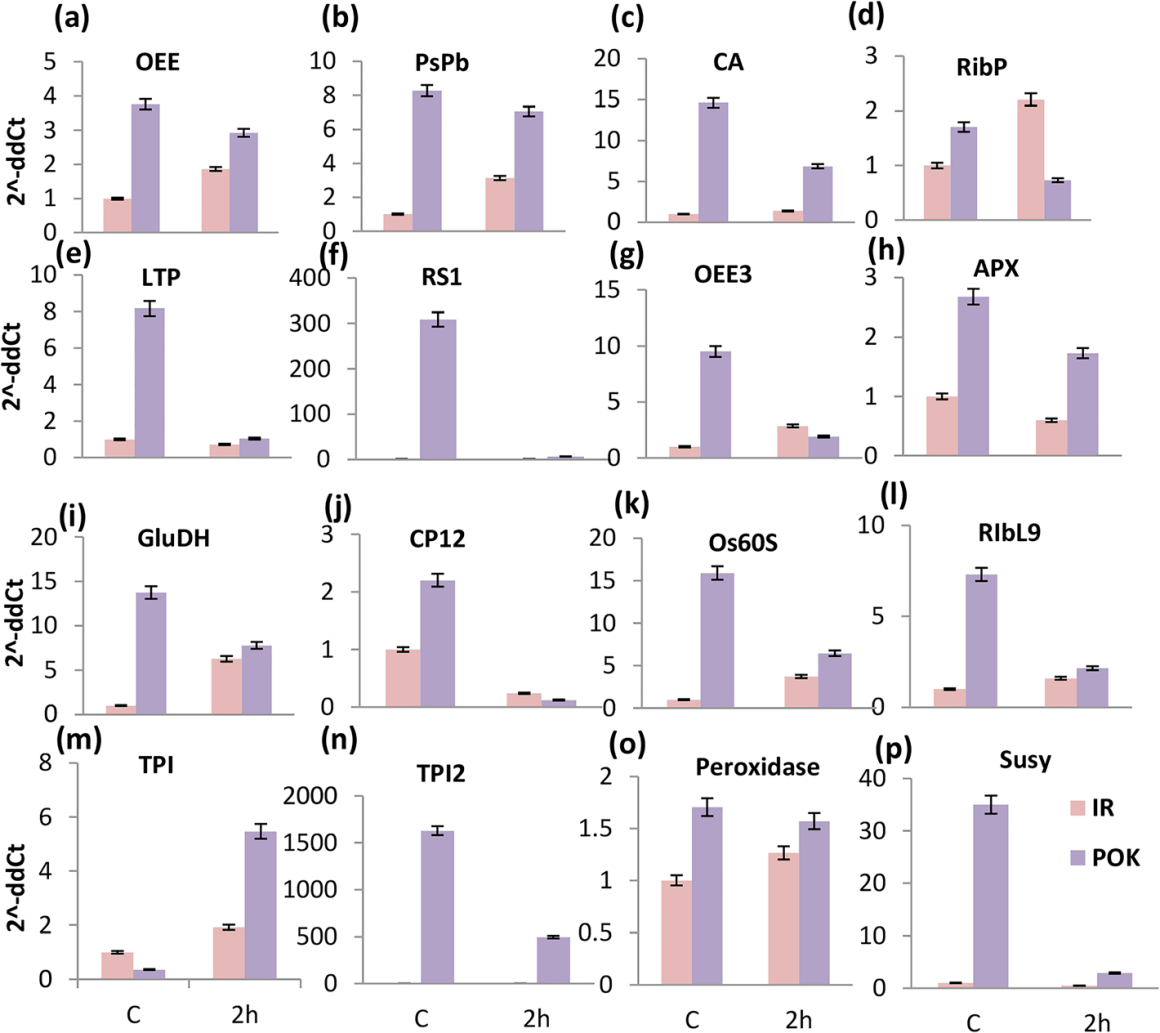
Salinity-regulated transcript profile of genes encoding selected proteins obtained from the proteome study. Histograms show fold change in expression in shoot (**a-l**) and root tissues (**m-p**) obtained using qRT-PCR. Genes used for the analysis were identified through iTRAQ proteome studies. Expression values have been calculated relative to non-stressed controls of IR64, taken as 1

In order to investigate the correlation between the transcript and protein profiles of differentially expressed proteins, we measured the transcript levels of few selected proteins under both control and stress conditions in Pokkali and IR64. Twelve proteins obtained from shoot proteome and four from root proteome were selected for this analysis. Proteins selected from shoot proteome primarily belonged to three major functional categories viz. protein metabolic process, photosynthesis and stress response as shown in Fig. 4a. Proteins such as, oxygen-evolving enhancer protein (OEE) 1, (PsbP), lipid transfer protein (LTP) - Protease inhibitor/seed storage, salt stress root protein RS1 and Ribosomal L9 (Rib L9) exhibited enhanced transcript levels under control as well as stress conditions in Pokkali with respect to IR64, much like their protein levels (Fig. 6 a-l). Other proteins such as, Calvin cycle protein CP12 (chloroplast protein), oxygen-evolving enhancer protein (OEE3) and ribosomal protein (RibP), exhibited an increase in their transcript levels in Pokkali under control conditions in correlation with their protein levels but under stress, though relative expression levels of these proteins remained higher in Pokkali vs. IR64, their transcript profile indicated that transcript accumulation was more in IR64 than Pokkali at 2 h of stress (Fig. 6 d,g,j). Ascorbate peroxidase (APX) was found to be the only protein whose transcript and protein levels showed positive correlation in their expression pattern at both control and stress conditions. Further, glutamate dehydrogenase (GluDH) protein levels though were not detectable under control conditions but transcript profile showed higher accumulation in Pokkali under control conditions. Under stress, both protein and transcript abundance of GluDH was more in Pokkali viz. IR64. Another protein, ribosomal protein (Rib L9) was is detected only under control conditions at protein level but transcripts accumulated under both control and stress conditions in Pokkali. A carbonic anhydrase (CA), was found to show increased transcript accumulation under both control as well as stress conditions in Pokkali with respect to IR64 but by contrast, its protein levels were less in Pokkali than IR64 under control conditions (Fig. 6 a-l).

Four proteins including, two triose phosphate isomerases (TPI), a sucrose synthase (Susy) and a peroxidase, obtained from the root proteome analysis were also studied for their transcript alterations in Pokkali and IR64. The transcript accumulation pattern of TPI matched its protein expression profiles under both control and stress conditions, however sucrose synthase exhibited an opposite transcript accumulation pattern in comparison to its protein profile under control conditions in Pokkali. Under salinity stress, however both transcript and protein levels of sucrose synthase were found to increase in Pokkali in comparison to IR64 (Fig. 6 m-p). Another candidate, peroxidase, showed an increase in transcript levels under both control and stress conditions in Pokkali even though its protein levels could not be detected under control conditions (Fig. 6 m-p). Overall, we could observe the differential pattern of gene expression with some genes showing correlations between their protein and transcript profiles.

### Protein-protein interaction network among differentially expressed proteins of Pokkali

**Fig. 7:**
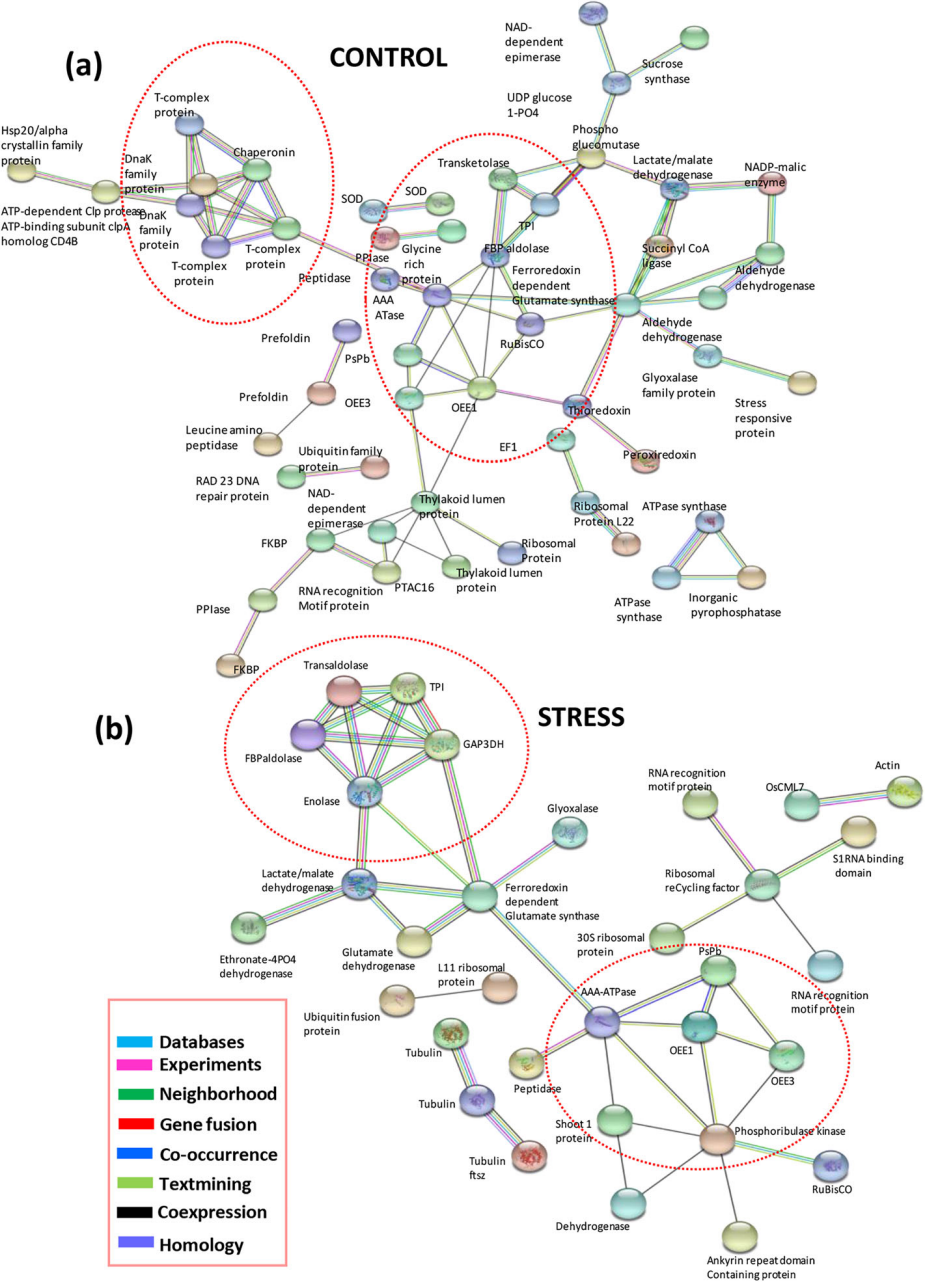
Protein-protein interaction network of differentially expressed proteins of Pokkali under control (**a**) and stressed (**b**) conditions. Network was developed using STRING software (https://string-db.org/). Circles show the major clusters of interacting proteins. Colored lines between the proteins indicate the various types of interaction evidence as denoted in the left corner

To predict the relationship among the differentially expressed proteins, two protein-protein interaction (PPI) networks were created using the differential Pokkali proteome which comprised of both root and shoot proteins. The analysis was carried out using STRING web tool and provided an overview of the differential protein networks operating in Pokkali w.r.t IR64 under control and stress conditions (Fig. 7 a, b). Of the 126 proteins differentially expressed under control conditions in Pokkali root and shoot tissues, 54 proteins were depicted in the network which showed interactions with each other and formed major clusters (Fig. 7a). The photosynthetic protein cluster comprising OEE1, OEE3, PsbP, thylakoid lumen protein and RuBisCo formed a major module which interacted with the network of metabolic enzymes involved in TCA/calvin cycle such as, transketolase and FBP aldolase (Fig. 7a). Further, stress-responsive genes such as superoxide dismutase, peroxidase, thioredoxin, and glyoxalase were also included in this interaction module, being connected with the above two networks of proteins. Notably, a chaperone protein network was also enriched under control conditions however, under stress, out of the 71 differentially expressed proteins in Pokkali shoot and root tissues, 32 were depicted in the network (Fig. 7b). The protein-protein interaction network of stressed Pokkali proteome retained the photosynthesis-related protein module and included glycolysis/TCA cycle protein network as well (Fig. 7b). In addition to this, proteins involved in RNA binding and translation formed another module, which was not seen under control conditions.

## Discussion

Salinity stress is an important constraint for agriculture affecting food production worldwide. So far, many studies have been undertaken to decipher the intricate processes operating under stress but still more efforts are needed to elucidate the components of stress response and adaptation. In the present work, we have studied the proteomes of Pokkali and IR64 under non-stress conditions as well as in response to short-term salinity stress using iTRAQ. We specifically chose an early, short duration stress (2 h) for our study, as we believe that some important changes needed for stress adaptation occur during this initial phase and it is during this time that the fate of plant survival is decided. However, it should not mean that later stages of salinity stress are not crucial for plant survival as recovery responses also need to be very efficient to help plants emerge from the stress. In the present study, our focus was to investigate initial phase of salinity response, which is in fact, a state of osmotic shock for the plants wherein, plants suffer more from the detrimental effects of changes in osmolarity rather than the accumulation of Na^+^ ions. Na^+^ accumulation contributes to ion toxicity at much later stages of the salinity response. Further, we have also investigated non-stress conditions to get insights into the pre-existing differences in the proteome of the two genotypes.

Our initial experiments demonstrated that Na^+^ accumulated in the shoot and root of Pokkali and IR64 seedlings after 2 h of salinity treatment as an indication towards the initiation of the salinity stress, but the increase was found to be more in shoots in IR64 whereas Pokkali showed more Na^+^ accumulation in roots. The capacity to exclude sodium from the shoot is usually an important determinant of salt tolerance in plants (Garthwaite et al. 2005; Kumari et al. 2009). In this context, Na^+^ accumulation pattern in potato varieties suggested a correlation between Na^+^ accumulation and stress tolerance, where the salt-sensitive potato variety Mozart was found to show higher sodium accumulation in leaves than roots and stem than the tolerant Desiree variety (Jaarsma et al. 2013). Though ion toxicity builds up during later stages of salinity stress, but even at 2 h, we could observe a similar pattern of Na^+^ accumulation in the genotypes, being higher in the shoot of salt-sensitive IR64 rice as compared to salt-tolerant Pokkali. Further, we detected higher levels of H_2_O_2_ in roots of Pokkali as compared to IR64 under stress conditions. Higher ROS levels in the system are usually an indicator of oxidative stress but higher levels may also confer ability for the constitutive activation of defense pathways that in turn keeps the tolerant cultivars prepared for adaptation to salt stress conditions (Kaur et al. 2016). On similar lines, a study conducted in salt-sensitive and salt-tolerant varieties of rice have revealed higher H_2_O_2_ and lower superoxide levels in the salt-tolerant varieties as compared to the sensitive ones (Kaur et al. 2016).

Pokkali and IR64 protein profiles revealed the expression-related differences in the two genotypes even under non-stress conditions. Eighty-six proteins were found to be significantly different in their expression pattern in Pokkali shoot tissues with respect to IR64 under control conditions. In roots, around 40 such proteins were identified to be possessing different expression levels in Pokkali and IR64. Importantly, these proteins were enriched in photosynthetic and protein metabolism-related functions in the shoot. For instance, photosynthesis related proteins such as Oxygen-evolving enhancer protein 1, PsbP, thylakoid lumenal 16.5 kDa proteins were present at higher levels in Pokkali than IR64 under non-stress conditions. In agreement, we have previously shown that Pokkali possesses higher photosynthetic rate than IR64 under non-stress conditions (Lakra et al. 2018). Further, proteins related to stress response such as, superoxide dismutase, ascorbate peroxidase, peptidyl-prolyl cis-trans isomerases, glyoxalase II and thioredoxin were also found to be enriched in Pokkali under non-stress conditions. This is in agreement with the previously reported higher activity of antioxidant machinery in Pokkali in comparison to IR64 under non-stress conditions such as, of superoxide dismutase, glutathione peroxidase, glutathione reductase, glyoxalases and catalase (El-Shabrawi et al. 2010; Lee et al. 2013). In addition, other proteins such as LTPs (Lipid transfer proteins), coproporphyrinogen III oxidase, phytocyanin related protein Pn14, NDPK1, HMG (high mobility group) protein and ferredoxin-dependent glutamate synthase also showed a higher expression in Pokkali under control conditions. NDPKs, which are among the oldest known proteins, mainly function in maintaining the metabolic balance between NTPs and NDPs in cells, signal transduction, elongation of rice coleoptile and plant stress response (Hasunuma et al. 2003; Ryu et al. 2005; Dorion et al. 2006). Interestingly, we found around 27 proteins in the shoot proteome of Pokkali to be > 60-fold higher in expression as compared to IR64 which included several stress responsive candidates.

In root proteome, proteins related to metabolism were enriched under normal conditions indicating high metabolic activity in roots of Pokkali. Proteins such as, triose phosphate isomerase, malate dehydrogenase, succinyl-CoA ligase beta-chain, sucrose synthase and malic enzyme were present at higher levels in Pokkali in comparison to IR64 which may result in the production of higher levels of sugar in roots. In this context, it has been reported that the accumulation of total soluble sugars and sucrose occurs to higher levels in the leaves of salt-sensitive rice variety, Khao Dawk Mali 105 in comparison to salt-tolerant Pokkali both under non-stress and stress conditions (Pattanagul and Thitisaksakul 2008). However, authors did not measure sugar level in roots. Further, Pokkali accumulates starch instead of sucrose in response to stress which may be a way of partitioning sugars to avoid metabolic alterations in response to salinity stress (Pattanagul and Thitisaksakul 2008). Around 5 proteins in root proteome of Pokkali had > 60-fold abundance when compared to IR64.

Under salinity stress, levels of proteins related to photosynthesis such as, oxygen-evolving enhancer protein 1, PsbP and oxygen evolving enhancer protein 3 remained elevated in the shoots of Pokkali. Further, proteins such as, Ribulose bisphosphate carboxylase large chain 1 and Ribulose bisphosphate carboxylase/oxygenase activase, were also increased under stress, in agreement with the observed increased photosynthetic activity in Pokkali under stress (Lakra et al. 2018). In addition, other proteins such as ankyrin repeat domain protein 2, LEA, OsCML7, ABA/WDS induced protein, inorganic pyrophosphatase, carbonic anhydrase and LTP were also found to be highly expressed under stress. The ankyrin repeat domain is present in some inward rectifying channels in plants which are involved in the low-affinity K^+^ transport (Fox and Guerinot 1998). Further, LTPs transport cutin or wax to the plasma membrane as a protection against water loss. Cutin is in fact, one of the main components of the plant cuticle which functions as a barrier against water loss.

Notably, a glutamate dehydrogenase protein was also highly elevated in Pokkali under stress. This enzyme acts as an important link between TCA cycle and amino acids metabolism and appears to have a significant role in the provision of carbon skeleton under conditions of carbon limitation (Athwal et al. 1997). A similar increase in the levels of this protein has also been reported in salt-tolerant rice varieties, CSR-1 and CSR-3 in comparison to salt-sensitive Ratna and Jaya varieties (Kumar et al. 2000). Another protein CDGSH iron sulfur protein (also referred to as mitoNEET) was found to be higher under stress in pokkali. CDGSH iron sulfur domains are generally located in the mitochondrial membranes and serve as transport channels for electron gradient regulation and iron transport (Lin et al. 2011). These proteins play a key role in modulating maximal capacity for electron transport and oxidative phosphorylation and are even involved in Fe-S cluster shuttling and in redox reactions.

Furthermore, a ferredoxin-dependent glutamate synthase protein showed 64-fold increase in Pokkali in comparison to IR64 under control conditions. It is known to be involved in glutamate biosynthesis in leaf and is also required for the re-assimilation of ammonium ions generated during photorespiration. In addition, we also found proteins such as, coproporphyrinogen III oxidase, lipid transfer protein (LTP), clathrin protein, RAD23, HMG transcription factor and ribosomal protein S11, to be highly expressed in Pokkali. The coproporphyrinogen III oxidase is involved in heme and chlorophyll biosynthesis and high mobility group (HMG) proteins play key functions in replication, transcription and nucleosome assembly.

In roots, a few proteins which showed altered levels under stress included peroxidases and TPI. Both these proteins have been known to be important for stress response. In fact, TPI has been shown to be regulated by methylglyoxal (MG), a toxic byproduct of glycolysis whose levels increase under stress (Sharma et al. 2012). MG induces TPI activity, which by metabolizing triose sugars prevents accumulation of MG in the system. Hence, TPI plays an important role in stress alleviation in plants by limiting MG levels (Sharma et al. 2012). Expression pattern of few proteins obtained from this analysis correlated well with those of our previous study. Proteins such as, oxygen-evolving enhancer protein 1, ribulose bisphosphate carboxylase large chain precursor, PsbP, ribose-5-phosphate isomerase A, superoxide dismutase, etc. were found through both iTRAQ (present study) and two-dimensional gel electrophoresis (Lakra et al. 2018), to be induced at 2 h in response to salinity stress. Determination of transcript levels of few proteins obtained from the present analysis revealed correlation in the transcript and protein expression pattern of some proteins.

Overall through this study, we could get some important insights into the differences in proteomes of salt-tolerant Pokkali and salt-sensitive IR64 under both normal and stress conditions. To summarize, Additional file 5: Figure S3 provides an overview of the differentially expressed proteins of Pokkali under both control and stressed conditions. The results indicated that most of these proteins were engaged in light reaction, redox related processes and stress responsive process. Our results suggest that Pokkali maintains a high activity of vital pathways such as, photosynthesis and of stress-responsive proteins, even under non-stress conditions which allow its survival and better adaptation under stress.

## Conclusions

Salinity stress poses a major risk to agriculture and hence, elucidating response mechanisms of plants to stress becomes necessary for understanding the stress adaptation dynamics and for raising tolerant crops to minimize the “yield gap”. To this end, we studied the response of salt-tolerant and salt-sensitive rice genotypes viz. Pokkali and IR64 to short-term salinity stress. Our studies conclude that Pokkali showed well preparedness to face stress conditions as the proteins otherwise, induced in response to stress in IR64, are naturally highly expressed in Pokkali even under control conditions, and upon encountering stress conditions, this pro-active stress machinery combats adverse conditions in a more efficient manner as compared to IR64.

## Material and methods

### Plant material and growth conditions

Seeds of Rice (*O. sativa* L. cv. IR64 and Pokkali) were surface sterilized and germinated for 48 h at 28 °C under hydroponic setup in a growth chamber as described earlier (Lakra et al. 2018). After 10 days of growth, seedlings were divided into two sets, of which one set was transferred to Yoshida medium containing 200 mM NaCl while the other set was used as control and so remained in the Yoshida medium (1972). After 2 h of NaCl treatment, root and shoot tissues of 20 seedlings of each group were harvested for proteome analysis, qRT-PCR analysis, and physiological studies.

### Determination of Na^+^ and K^+^ content

One hundred milligrams tissue (roots or shoots) harvested from control and NaCl treated plants, was digested in 0.1% HNO_3_ and the concentration of Na^+^ and K^+^ was recorded by AAS (atomic absorption spectroscopy) as described earlier (Kumar et al. 2009).

### Relative ion leakage

Ion leakage was measured as described by Bajji et al. (2002). Briefly, leaf tissues harvested from the control and NaCl treated plants were first washed with distilled water to remove any ions adhering to the surface. One hundred milligrams tissue was then dipped into the de-ionized water and incubated at 37 °C for 2 h, following which, electrical conductivity (E_1_) of the solution was measured using a conductivity meter (ELEINS, Inc., India). After measuring E_1_, samples were autoclaved for 15 min. Total conductivity (E_2_) was measured once the samples cooled down to room temperature. Relative electrical conductivity was calculated using the formula: Ion leakage percentage = E_1_/E_2_*100.

### Cell viability test

Cell viability of roots tissues was determined as described earlier by Sanevas et al. (2007). Fresh root samples were first stained with Evans Blue dye (0.25%) for 15 min at room temperature followed by washing in distilled water for 45 min to remove any surface-bound dye. Next, Evans Blue stain taken up by the dead cells was extracted at 55 °C for 1 h using 1% (*w*/*v*) SDS. Finally, absorbance was measured at 600 nm to determine the amount of Evans Blue uptake by the roots.

### Detection of hydrogen peroxide (H_2_O_2_) by DAB staining

For the detection of H_2_O_2_, roots of seedlings were incubated in 3,3-diaminobenzidine (DAB; 1 mg/ml concentration) staining solution for 2–4 h under the dark conditions followed by washing to remove the extra dye before viewing under microscope (Daudi and O’Brien 2012).

### Confocal microscopy for detection of sodium ions using CoroNa green dye

Root samples were visualized using CoroNa Green dye as described by Gupta et al. (2018). A confocal laser scanning microscope (Fluoview FV300; Olympus, Tokyo, Japan) with a 488-nm excitation and 505–525 nm emission wavelength was used. Equal photomultiplier tube (PMT) settings were used to visualize images which prevented artifacts for each sample set. Average fluorescence intensity was measured by subtracting the background of corresponding image for each sample.

### iTRAQ labeling, strong cation exchange fractionation and reverse phase nanoLC

Protein samples were processed for iTRAQ using the iTRAQ Reagents Multiplex kit (Applied Biosystems/MDS Sciex, Foster City, CA, USA). Twenty milligrams protein samples of IR64 and Pokkali obtained from either control or NaCl (200 mM) treated plants were labeled with different iTRAQ reagents. IR64 samples (control and NaCl-treated) were labeled with 114 and Pokkali (control and NaCl-treated) by 117 iTRAQ reagents. After labelling, control samples of both IR64 and Pokkali were pooled and similar pooling was done for NaCl-treated labelled samples. This was followed by vacuum-drying and ultimately samples were subjected to strong cation exchange (SCX) fractionation on the Agilent 1100 HPLC system using a PolySulfoethyl column (4.6 × 100 mm, 5 μm, 300 A). However, before SCX fractionation, labelled peptides were first desalted using a Strata-X 33 μm polymeric reversed phase column (Phenomenex) followed by resuspension in buffer containing 10 mM KH_2_PO_4_ in 10% acetonitrile, pH 3.0. After fractionation, peptides were eluted with a linear gradient of 0–400 mM KCl. Eight fractions were collected and again desalted on Strata-X columns.

For second dimension reverse phase nanoLC, fractions were loaded onto a C18 PepMap100 column, a 3 μm column (LC Packing) running on an Ultimate 3000 nano HPLC system (Dionex). Peptides were resolved with a gradient of 10–40% acetonitrile (prepared in 0.1% trifluoroacetic acid) and fractions were spotted using a ProBot robotic spotter (LC Packings) on the AnchorChip MALDI plates. The spots were analysed by 5800 MALDI TOF/TOF Analyzer.

### Data analysis

Spectral data was analysed using ProteinPilot™ 4.0 Software (AB Sciex) against the UniProt rice database. The database containing 2,88,134 protein sequences was used to extract peptide and protein data at > 95% confidence levels and high top one peptide rank filters. False discovery rate (FDR) was determined using Proteomics System Performance Evaluation Pipeline (PSPEP) feature of ProteinPilot™ software. For achieving high confidence identifications, target FDR threshold was set at 1%. Relative quantitation of proteins was based on the relative intensities of reporter ions released during the MS/MS peptide fragmentation. In order to determine the relative protein contents of the samples, only unique peptides for each identified protein were taken.

### Bioinformatics analysis

The identified proteins were annotated using Gene Ontology (GO) database (https://www.blast2go.com/) and assigned protein functions using the protein function databases, InterPro (http://www.ebi.ac.uk/interpro/) or Pfam (https://pfam.xfam.org//) and finally confirmed by MSU v7.0 rice database (http://rice.plantbiology.msu.edu/). Clustering was performed based on fold-induction expression values from control (Pok/IR64) and stress (Pok/IR64) samples using the Multi Experiment Viewer software (The Institute for Genomic Research). The data was clustered using Pearson correlation (Romijn et al. 2015). A PPI (Protein-Protein Interaction) network was constructed using STRING 10.5 tool with a confidence level of 0.7 (https://string-db.org). To analyze the metabolic and signaling changes in protein expression under control and stress conditions, a MapMan tool (http://mapman.gabipd.org/web/guest/mapman) (Thimm et al. 2004) was used.

### Real-time quantitative reverse transcription PCR

Total RNA was isolated, cDNA was prepared and qRT-PCR was performed as described earlier (Soda et al. 2013). Primers used for the analysis have been listed as Additional file 6: Table S3. The rice elongation factor (eEF1α) was used as a house-keeping gene for data normalization. For every sample, two biological replicates were used, each having three technical replicates (*n* = 6).

### Statistical analysis

All the data (from three replicates) from physio-chemical analysis, were subjected to ANOVA (analysis of variance) using the GraphPad InStat3 software. For iTRAQ, differentially expressed proteins with at least 1.5-fold change and *p* value < 0.05 were selected.

## Additional files

Additional file 1: **Figure S1.** Evan blue staining of the roots of Pokkali and IR64 seedlings in response to 2 h of salinity stress. (TIFF 314 kb)

Additional file 2: **Figure S2.** DAB staining of the roots of Pokkali and IR64 seedlings in response to 2 h of salinity stress. (TIF 10980 kb)

Additional file 3: **Table S1.** Highly differentially expressed proteins (>60 fold) in shoot and root tissues. (XLSX 18 kb)

Additional file 4: **Table S2.** Differentially expressed proteins in shoot tissues of Pokkali w.r.t IR64 which are commonly appearing under non stress and stress conditions. (XLSX 13 kb)

Additional file 5: **Figure S3.** Depiction of differentially expressed proteins on metabolic pathways using Mapman. Individual elements in the metabolic overview, stress response and redox overview are indicated by solid red rectangular boxes. Rectangular boxes indicate over-represented Mapman functional groups under control and stress conditions in Pokkali with respect to IR64. (TIFF 1525 kb)

Additional file 6: **Table S3.** List of RTPCR primers and their sequences (5'to 3') used in this study. (XLSX 10 kb)
