# Supplementary figures and images for: Mapping the ‘early salinity response’ triggered proteome adaptation in contrasting rice genotypes using iTRAQ approach
Source: Rice (N Y). 2019 Jan 30;12:3. doi: 10.1186/s12284-018-0259-5 (PMC6357216; doi:10.1186/s12284-018-0259-5)

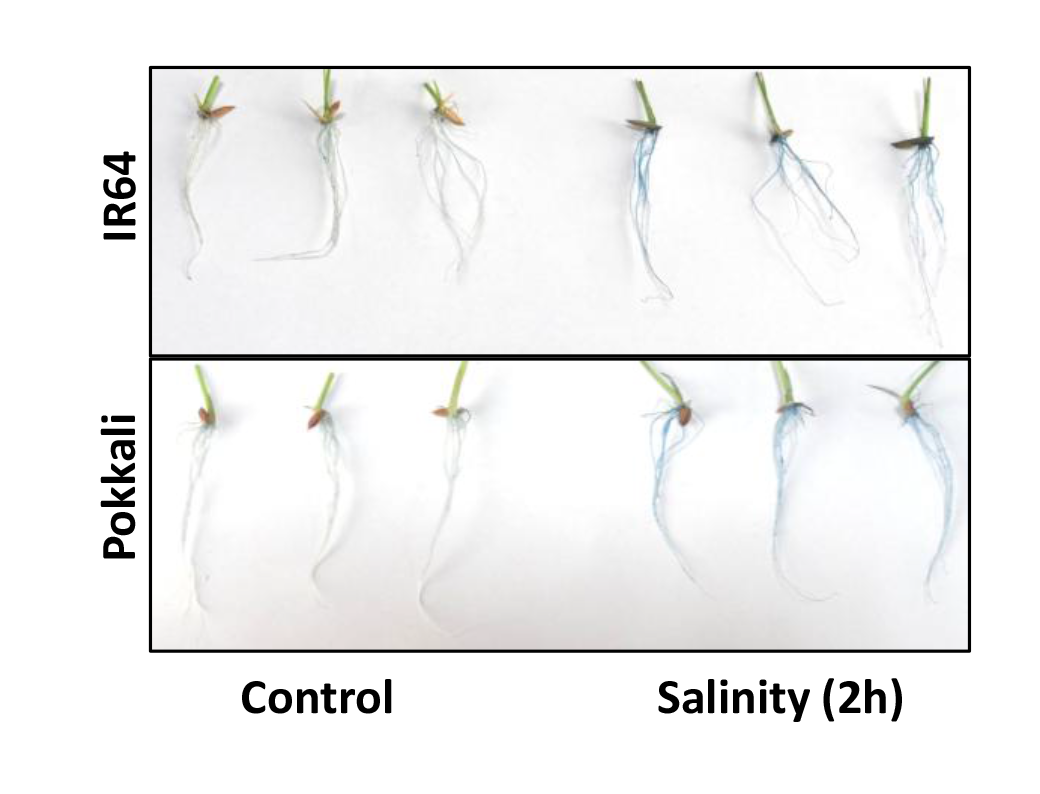

Supplement: Supplementary file 1 — Figure S1. Evan blue staining of the roots of Pokkali and IR64 seedlings in response to 2 h of salinity stress. (TIFF 314 kb) [file 12284_2018_259_MOESM1_ESM.tiff]

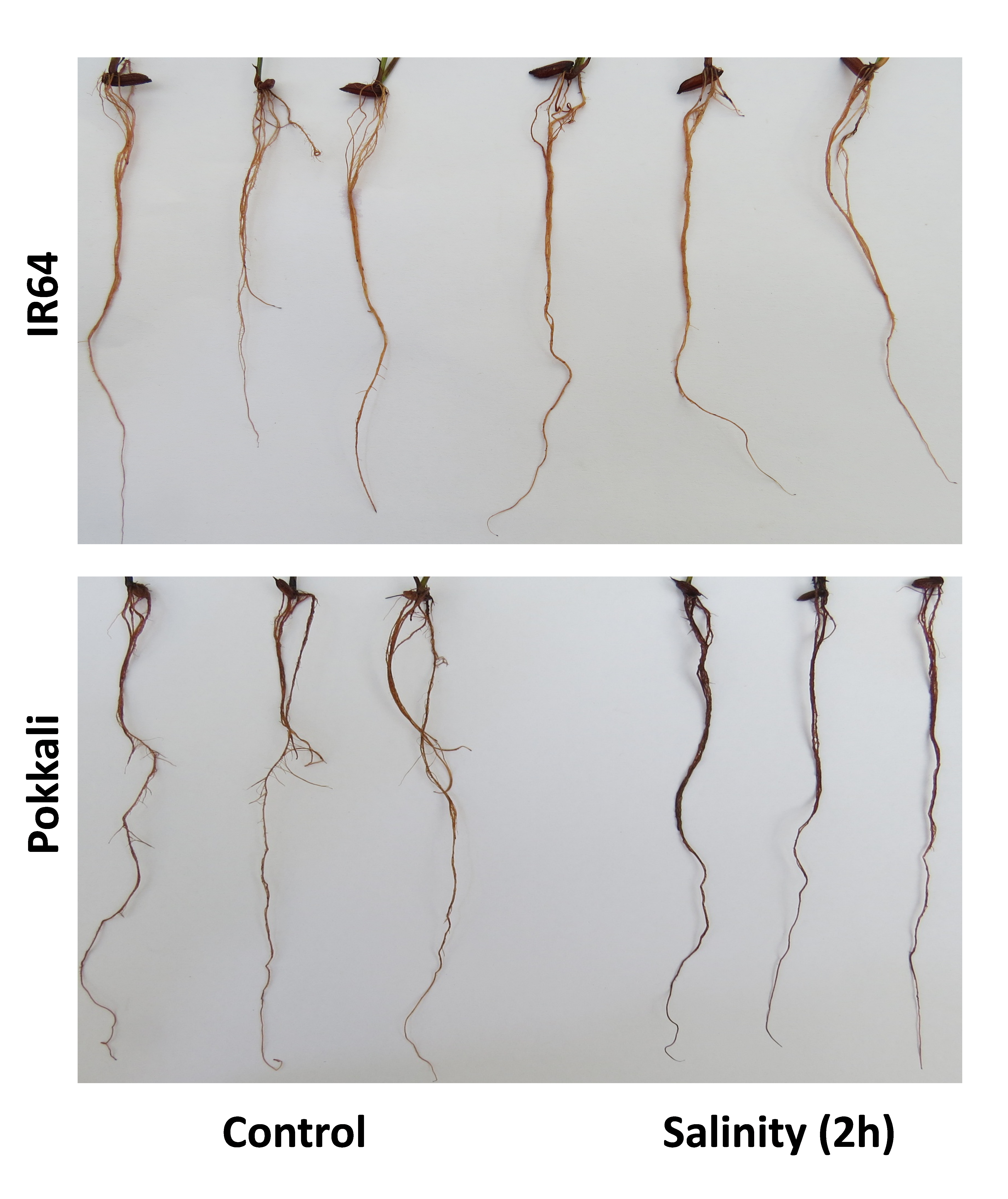

Supplement: Supplementary file 2 — Figure S2. DAB staining of the roots of Pokkali and IR64 seedlings in response to 2 h of salinity stress. (TIF 10980 kb) [file 12284_2018_259_MOESM2_ESM.tif]

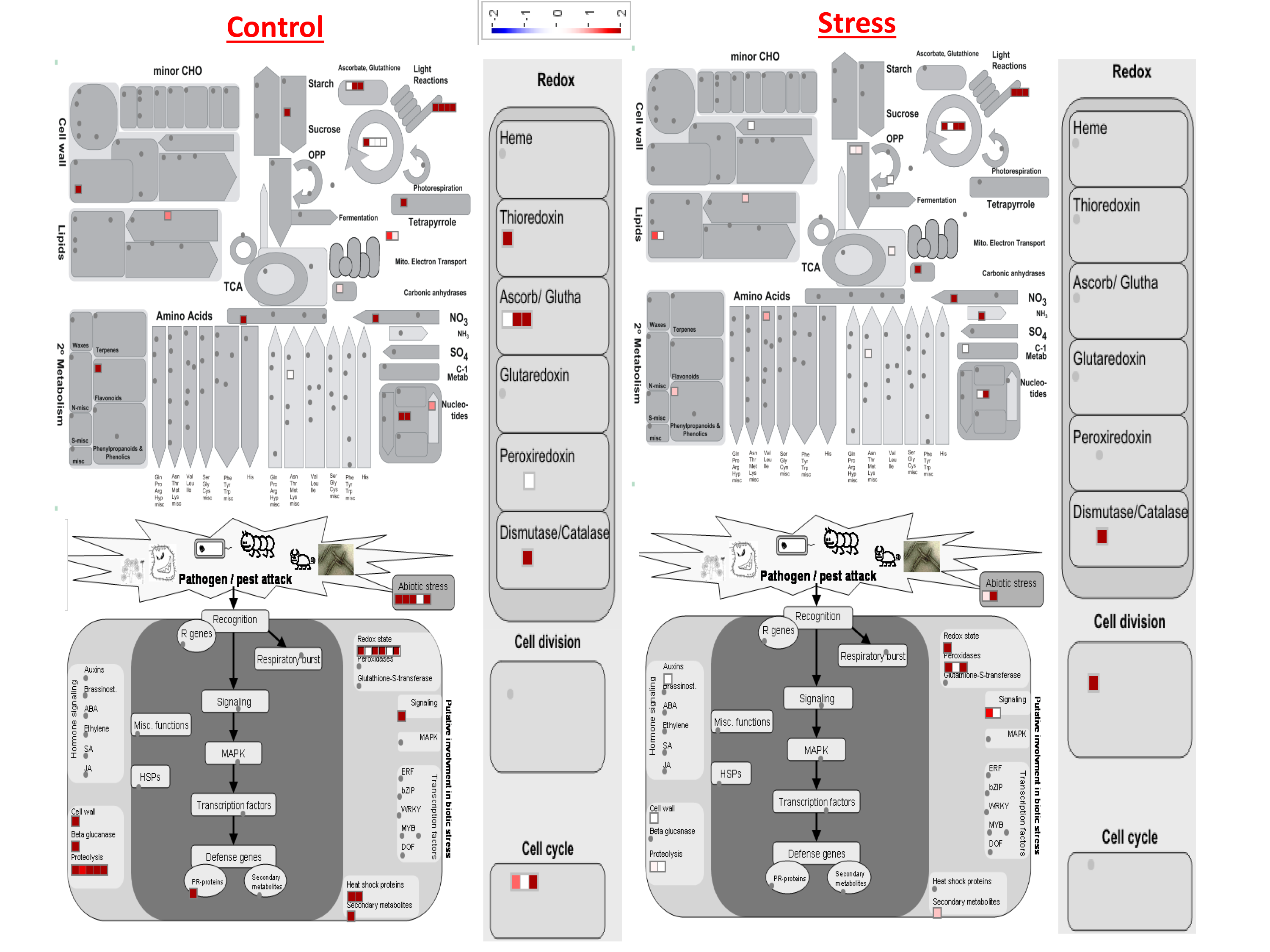

Supplement: Supplementary file 5 — Figure S3. Depiction of differentially expressed proteins on metabolic pathways using Mapman. Individual elements in the metabolic overview, stress response and redox overview are indicated by solid red rectangular boxes. Rectangular boxes indicate over-represented Mapman functional groups under control and stress conditions in Pokkali with respect to IR64. (TIFF 1525 kb) [file 12284_2018_259_MOESM5_ESM.tiff]
